# Comparison of Trihelix transcription factors between wheat and *Brachypodium distachyon* at genome-wide

**DOI:** 10.1186/s12864-019-5494-7

**Published:** 2019-02-15

**Authors:** Chengwei Wang, Yu Wang, Qi Pan, Shoukun Chen, Cuizhu Feng, Jiangbo Hai, Haifeng Li

**Affiliations:** State Key Laboratory of Crop Stress Biology for Arid Areas, College of Agronomy, Northwest A&F University, Yangling, 712000 China

**Keywords:** *Trihelix*, GT transcription factors, Wheat, *Brachypodium distachyon*

## Abstract

**Background:**

Plant Trihelix transcription factors, specifically bind to GT elements and play important roles in plant physiology and development. Wheat is a main cereal crop. *Brachypodium distachyon* is a close relative of wheat and has been described as a new model species for studying of grass functional genomics. Presently, little is known about wheat and *B. distachyon Trihelix* genes.

**Results:**

In 51 species, 2387 *Trihelix* genes were identified, including 80 wheat *Trihelix* genes and 27 *B. distachyon Trihelix* genes. Consistent with the results of previous studies, these genes were classified into five subfamilies: GT-1, GT-2, SIP1, GTγ, and SH4. Members of the same subfamily shared similar gene structures and common motifs. Most *TaGT* and *BdGT* genes contained many kinds of *cis*-elements, such as development-, stress-, and phytohormone-related *cis*-acting elements. Additionally, 21 randomly selected *TaGT* genes were mainly expressed in the roots and flowers, while the expression of 19 selected *BdGT* genes was constitutive. These results indicate that the roles of *Trihelix* genes in wheat and *B. distachyon* might have diversified during the evolutionary process. The expression of the most selected *TaG*T and *BdGT* genes was down-regulated when exposed to low temperatures, NaCl, ABA, and PEG, implying that *TaGT* and *BdGT* genes negatively respond to abiotic stress. On the contrary, the expression of some genes was up-regulated under heat stress.

**Conclusions:**

*Trihelix* genes exist extensively in plants and have many functions. During the evolutionary process, this gene family expanded and their functions diversified. As a result, the expression pattern and functions of members of the same family might be different. This study lays a foundation for further functional analyses of *TaGT* and *BdGT* genes.

**Electronic supplementary material:**

The online version of this article (10.1186/s12864-019-5494-7) contains supplementary material, which is available to authorized users.

## Background

Trihelix transcription factors (TFs), also named GT TFs, contain three tandem helices (helix-loop-helix-loop-helix) [1, 2]. The nomenclature GT originated from the first identified *Trihelix* gene pea (*Pisum sativum*) GT-1, which binds specifically to the light-induced gene *rbcS-3A* [2]. *Trihelix* genes extensively exist in plants. For example, there are 30 genes in *Arabidopsis thaliana* [3], 31 in rice [4], 52 in *Brassica Rapa* [5], 36 in tomato [6] and 20 in *Chrysanthemum morifolium* [7]. According to their sequence similarities, Trihelix TFs were divided into five clades: GT-1, GT-2, SIP1, GTγ, and SH4 [3].

*Trihelix* genes play extensive roles in plant growth and response to environmental stress. *GT-4*, a clade member of GT-1 in *Arabidopsis*, plays an positive role in salt tolerance by directly binding to the Cor15A promoter [8]. Another clade member of GT-1, *Solanum habrochaites* ShCIGT functions in plant development and response to stress. Transgenic tomato overexpressing *ShCIGT* exhibited dwarf phenotype and enhanced tolerance to cold and drought [9].

Distinct from other GT TFs, clade members of GT-2 have two DNA-binding domains [3]. In *Arabidopsis,* the GT-2 gene *PETAL LOSS* (*PTL*) functions in flower development [10]; *GT-2-LIKE1* (*GTL1*) represses root hair growth [11]; *AtGT2L* encodes a Ca2+/CaM-binding nuclear transcription factor and is involved in the response to cold and salt [12]. In wheat, *TaGT2L1* negatively regulates drought tolerance and stomatal development [13]. In *B. distachyon*, the transcription factor BdTHX1 likely plays an important role in the biosynthesis of Mixed-linkage glucan (MLG) by regulating the expression of *BdCSLF6* (a *B. distachyon* Cellulose synthase-like gene) and *BdXTH8* (a *B. distachyon* cellulose synthase-like H gene) [14].

The first SIP1 member, NtSIP1 (for *Nicotiana tabacum* 6b–interacting protein 1), was identified in tobacco and seemed to function in the proliferation of plant cells, through association with 6b protein (encoded by the T-DNA of Agrobacterium) [15]. *Arabidopsis* SIP1 member *ASIL1* acts as a temporal regulator of seed filling by repressing the expression of master regulatory genes *LEC2*, *FUS3*, *ABI3* and other related genes [16]. In *Brassica napus, the expression of BnSIP1–1* is induced by ABA and abiotic stress. Transgenic *Brassica napus* lines overexpressing this gene improved the seed germination under osmotic pressure, salt, and ABA treatments [17].

Rice genes *OsGTγ-1*, *OsGTγ-2* and *OsGTγ-3* were the first clade members of GTγ to be identified. Expression of *OsGTγ-1* is up-regulated under salt stress, with transgenic rice plants over-expressing *OsGTγ-1* showing enhanced salinity tolerance [18]. In addition to its role in abiotic stress tolerance, *GTγ* also plays important functions in the response to biotic stress. For example, *TuGTγ-3* functions in the resistance to stripe rust in *Triticum urartu* [19].

*Arabidopsis* SH4 gene *ASR3* was reported to regulate the expression of genes related to immunity (so called immune genes) [20]. Rice Shattering 1(*SHA1*) gene plays an important role in cell separation. Mutation in the trihelix domain results in the elimination of seed shattering [21]. Another clade member of SH4, *GhGT29* (*Gossypium hirsutum*) might be involved in the regulation of stress resistance-related genes [22].

Common wheat (*Triticum aestivum*) is an important cereal crop. *B. distachyon* is a new model species of grass and has a close genetic relationship with common wheat [23]. As genome sequencing of wheat and *B. distachyon* has been completed [24, 25], it is urgent to elucidate the functions of important genes. However, to date, only the genes *TaGT* and *BdGT* have been functionally analyzed [13, 14]. In this study, *TaGT* and *BdGT* were analyzed at the genome level. A comparison between *Trihelix* genes in wheat and *B. distachyon* was further performed. Our results lay a foundation for further functional elucidation.

## Results

### Identification of wheat and *B. distachyon* Trihelix TFs

A total of 80 TaGT and 27 BdGT TFs were identified. Most of them (70 *TaGT* and 23 *BdGT* genes) were verified by ESTs (Additional files 1 and 2). All predicted wheat *Trihelix* genes were named from *TaGT1-A* to *TaGT36-D*, based on their chromosomal order and genomic homology (Additional file 1). *BdGT* genes were renamed from *BdGT1* to *BdGT27* based only on their chromosomal order (Additional file 2). They were distributed on chromosomes unevenly (Additional files 3 and 4). Among 80 *TaGT* genes, 51 genes constituted 17 sets. Every set included three homologous genes in A, B, and D sub-genomes respectively; other 20 genes formed 10 sets. Every set has homologous genes. The parameters of these GT proteins were predicted. The length of TaGT proteins ranged from 129 (TaGT16-D) to 800 amino acids (TaGT35-D); the PI ranged from 4.8 (TaGT16-D) to 10.39 (TaGT1-A); the molecular weight varied form 1.2 to 85.1 kDa (Additional file 1). In 27 BdGT proteins, the length ranged from 242 (BdGT10) to 875 amino acids (BdGT15); the PI ranged from 5.27 (BdGT14) to 10.05 (BdGT27), and the molecular weight was between 27.6 and 96.6 kDa (Additional file 1). Noticeably, most TaGT and BdGT TFs were hydrophilic proteins (Additional files 1 and 2).

### Sequence alignment and phylogenetic analyses of Trihelix TFs

To further analyze wheat and *B. distachyon* Trihelix TFs, multiple sequence alignment was performed. Results showed that GT domains were highly conserved (Additional file 5). There were invariant amino acids in the three tandem helices, including tryptophan (W) and leucine (L) in Helix1; tryptophan (W), valine (V) and glycine (G) in Helix2; and glutamine (Q), cysteine (C) and tyrosine (Y) in Helix3 (Additional files 5 and 6). In addition, many hydrophobic amino acids (tryptophan, leucine, valine, tyrosine) were found in TaGT and BdGT proteins. Consistent with these results, most TaGT and BdGT TFs were hydrophilic proteins. These hydrophobic amino acids may affect the protein structure and functions [3].

Furthermore, we identified *Trihelix* genes in 51 species, including algae species, seed and un-seed plants. Totally, 2387 genes were identified and grouped into GT-1, GT-2, SIP1, GTγ, and SH4 (Additional file 7, Fig. 1). Similar to seed plants, some non-seed species had members in all clades GT-1, GT-2, SIP1, GTγ, and SH4, while some algae species only had one or two sub-group genes. For example, *Dunaliella salina* and *Chlamydomonas reinhardtii* only had SIP1 clade genes; *Coccomyxa subellipsoidea* and *Micromonas pusilla* only had clade members of SIP1 and GT-2. The number of *Trihelix* genes was greatly variable among 51 plant species, ranging from two to three in algae to 121 in *Panicum virgatum*. SIP1 is the largest subfamily, and was found in all selected species, whereas the subfamily SH4 appeared latest. These results indicate that the *Trihelix* gene family is an ancient gene family that might have undergone gene expansion during evolution.

**Fig. 1:**
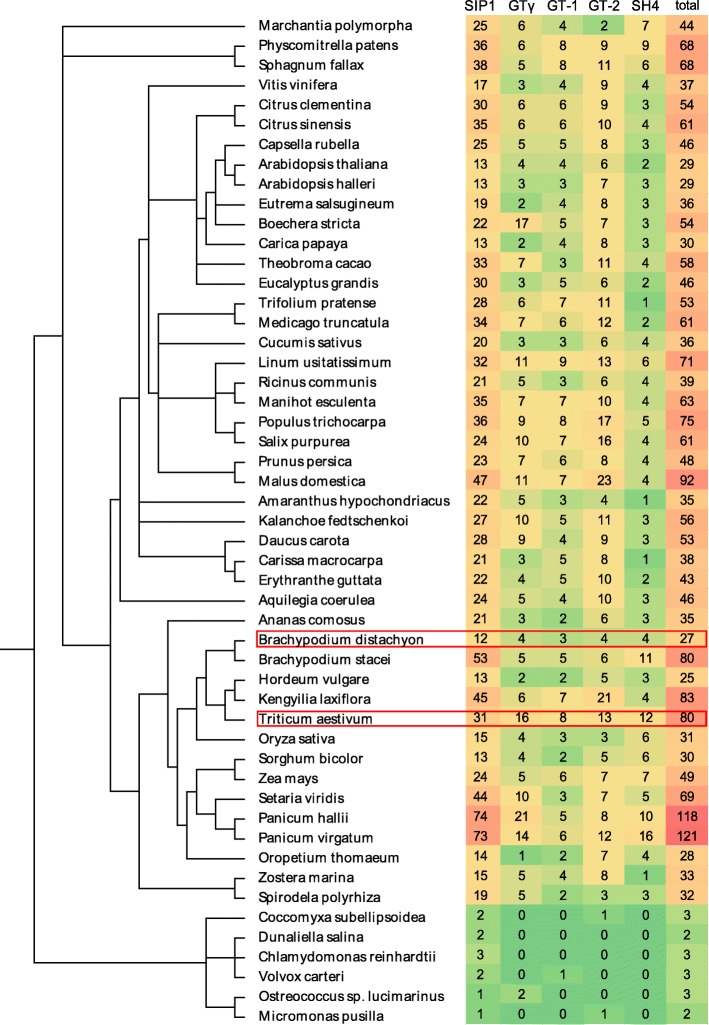
Phylogenetic relationships between the 51 plant species investigated in this study. The total number of Trihelixs and each groups identified in each plant species was presented on the right

**Fig. 2:**
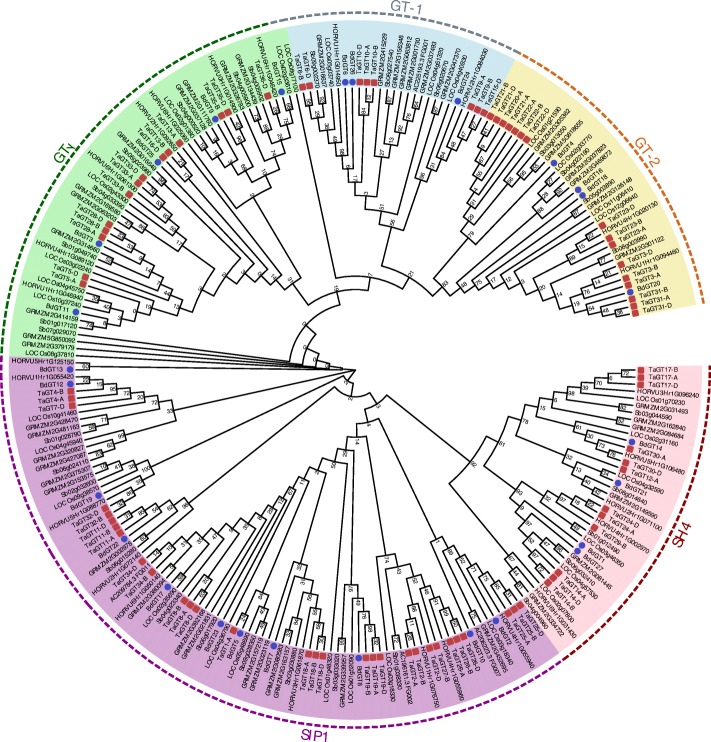
Phylogenetic tree of Trihelix proteins. The five different subfamilies were indicated by different colors. Trihelix proteins of TaGT and BdGT TFs were indicated by red and blue circles respectively

To study the evolutionary relationships of *Trihelix* genes in Gramineae plants, un-rooted Neighbor-joining (NJ) and Maximum Likelihood (ML) phylogenetic trees were constructed using 249 putative Trihelix proteins of wheat, *B. distachyon,* maize, rice, sorghum, and barley (Additional file 8). Results of these two trees were consistent (Additional file 9; Fig. 2).

### Gene structure and conserved motifs of *Trihelix* genes

**Fig. 3:**
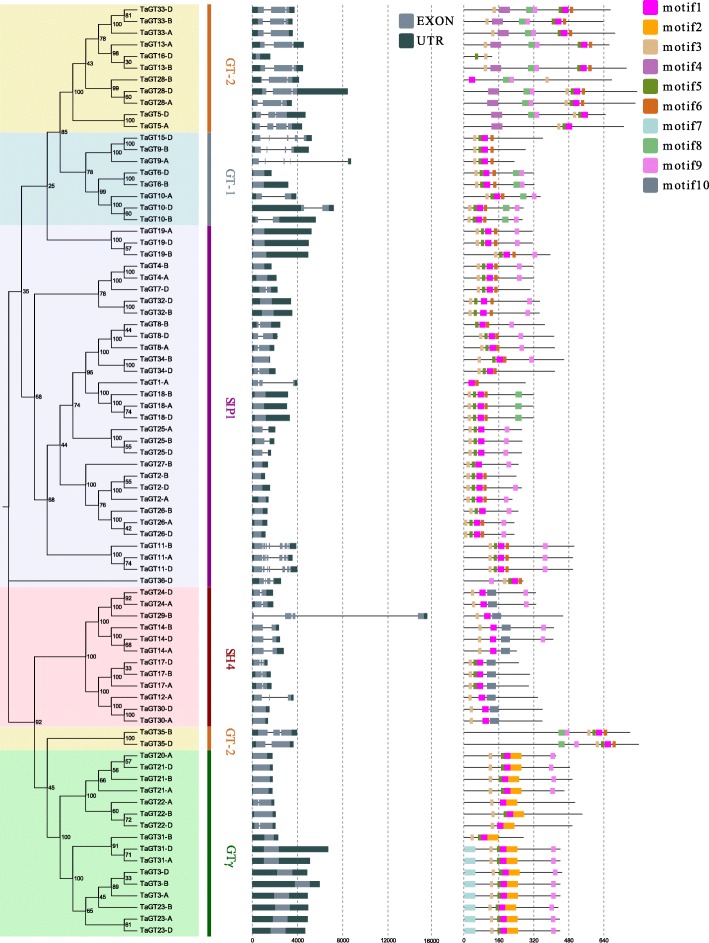
Gene structures and motifs of TaGT proteins. In the gene structure part, black boxes represent UTRs, grey boxes represent exons, and gray lines represent introns. In the motif part, the boxes in different color represent different motifs, and the grey lines represent un-conserved sequences

**Fig. 4:**
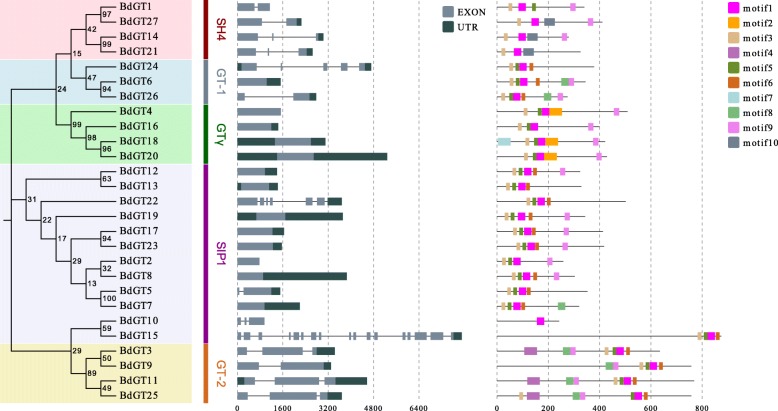
Gene structures and motifs of BdGT proteins. In the gene structure part, black boxes represent UTRs, grey boxes represent exons, and gray lines represent introns. In the motif part, the boxes in different color represent different motifs, and the grey lines represent un-conserved sequences

Gene structure is helpful to understand the evolution of genes. As shown in Fig. 3, the exon number of *TaGT* genes ranged from 1 to 8. Except for *TaGT22-A* and *TaGT22-D* with two exons, other GTγ genes only had one exon. In *B. distachyon*, the exon number of most *BdGT* genes ranged from 1 to 7. One exception was *BdGT16*, which had 16 exons. Similar to *TaGT* genes, *BdGT* genes in the subfamily GTγ had one exon (Fig. 4). In general, members of the same family might share similar gene structure.

Furthermore, we predicted the conserved motifs of TaGT and BdGT proteins. A total of 10 motifs were identified (Figs. 3 and 4). Almost all TaGT proteins had motif 1 and motif 3, except for TaGT1-A (without motif 3) and TaGT16-D (without motif 1). Motif 7 and 10 was only found in some clade members of GTγ and in some proteins of SH4, respectively. Similarly, motif 1 and motif 3 were identified in all BdGT proteins, while motif 7 and 10 only appeared in BdGT18 which belonged to the subfamily GTγ and SH4, respectively. These results show that the gene structure and motifs of TaGT and BdGT TFs were conserved. As a result, members with conserved motif compositions and similar gene structures were divided into the same groups. Our phylogenetic analyses results and previous studies clearly showed the reliability of this classification [26, 27].

### *Cis*-elements and gene ontology (GO) annotation

The *cis*-regulatory elements of promoter regions are related to gene expression patterns and functions [28]. Martin et al. showed that regulatory sequences were located 1500-bp upstream of the start codon [29]. Therefore, we analyzed the 1500-bp promoter region of *TaGT* and *BdGT* genes. A total of 249 and 233 kinds of *cis*-acting elements were detected in TaGT and BdGT gene promoters, respectively. Among them, 216 were identical (Additional files 10 and 11).These results further showed that *TaGT* and *BdGT* genes were conserved to some extent. Most cis-elements were related to plant growth/development and abiotic stress response (Additional file 12). *Cis*-elements related to growth/development are, among others, associated with light response (SORLIP1AT, GATABOX, GT1CONSENSUS, IBOXCORE, and SORLIP2AT), seed expression (CAATBOX1, EBOXBNNAPA, MYCCONSENSUSAT, and SEF4MOTIFGM7S), leaf expression (DOFCOREZM, GT1CONSENSUS, MYBCORE, MYCCONSENSUSAT and RAV1AAT), shoot expression (GT1 CONSENSUS, MYBCORE, RAV1AAT and IBOX CORE), root expression (RAV1AAT and ROOTMOTIFTAPOX1), pollen expression (GTGANTG10 and POLLEN1LELAT52). *Cis*-elements related to abiotic stresses are, among others, involved in ABA response (MYCCONSENSUSAT and DPBFCOREDCDC3), gibberellin (GA) response (WRKY71OS), salicylic acid (SA) response (WBOXATNPR1), heat response (CCAATBOX1), salt stress (GT1GMSCAM4), drought response (CBFHV and ACGTATERD), cold response (MYCCONSENSUSAT and CBFHV), oxygen response (CURECORECR and CURECORECR), wounding response (WBOXNTERF3). Trihelix promoters such as GT1GMSCAM4 (pathogen-related), NODCON2GM (root nodules related), WBOXATNPR1 (disease resistance related), and OSE2ROOTNODULE (root nodules related) also contained *cis*-elements related to biotic stresses. Identification of such high numbers of cis-elements further implies the extensive functions of *Trihelix* genes.

29 *cis*-elements were found in more than 80% *TaGT* and *BdGT* genes (Additional file 13). Among them, 6 are involved in light response, 15 are related to plant tissue-specific expression and 13 respond to stress. We also investigated the conservation of *cis*-elements at the sub-family level. In wheat, clade members of GT-1, GT-2, and SH4 had 9, 2 and 3 of the same *cis*-elements, respectively and the same 10 *cis*-elements in all *TaGTs* (Additional file 14). In *B. distachyon*, members of GT-1, GTγ and SH4 had corresponding numbers of 7, 3 and 3 of the same cis-elements, respectively, and 8 *cis*-elements in all *BdGTs* (Additional file 14).

In order to predict the functions of *TaGT* and *BdGT* proteins, gene ontology (GO) annotation analyses were performed. A total of 19 distinct functional groups were identified: 10 involved in biological processes, 6 involved in cellular components and 3 involved in molecular functions (Additional file 15). In wheat, GO classifications of ‘binding’ (54 proteins, 66.67%), ‘biological regulation’ (37, 45.68%), ‘cell’ (30, 37.04%), ‘cell part’ (30, 37.04%), and ‘organelle’ (30, 37.04%) were dominantly attributed. Similarly, BdGT proteins were mainly grouped in the GO categories of ‘binding’ (19 proteins, 70.37%), ‘biological regulation’ (9, 33.33%), ‘cell’ (7, 25.93%), ‘cell part’ (7, 25.93%), and ‘organelle’ (7, 25.93%). Among these GO categories, *TaGT* and *BdGT genes* shared similar proportions of distributions. On one hand, these results indicate the multi-functions of *TaGT* and *BdGT* genes; on the other hand, they further suggest functional conservation between *TaGT* and *BdGT* TFs.

### Homologous gene pairs and synteny analysis

**Fig. 5:**
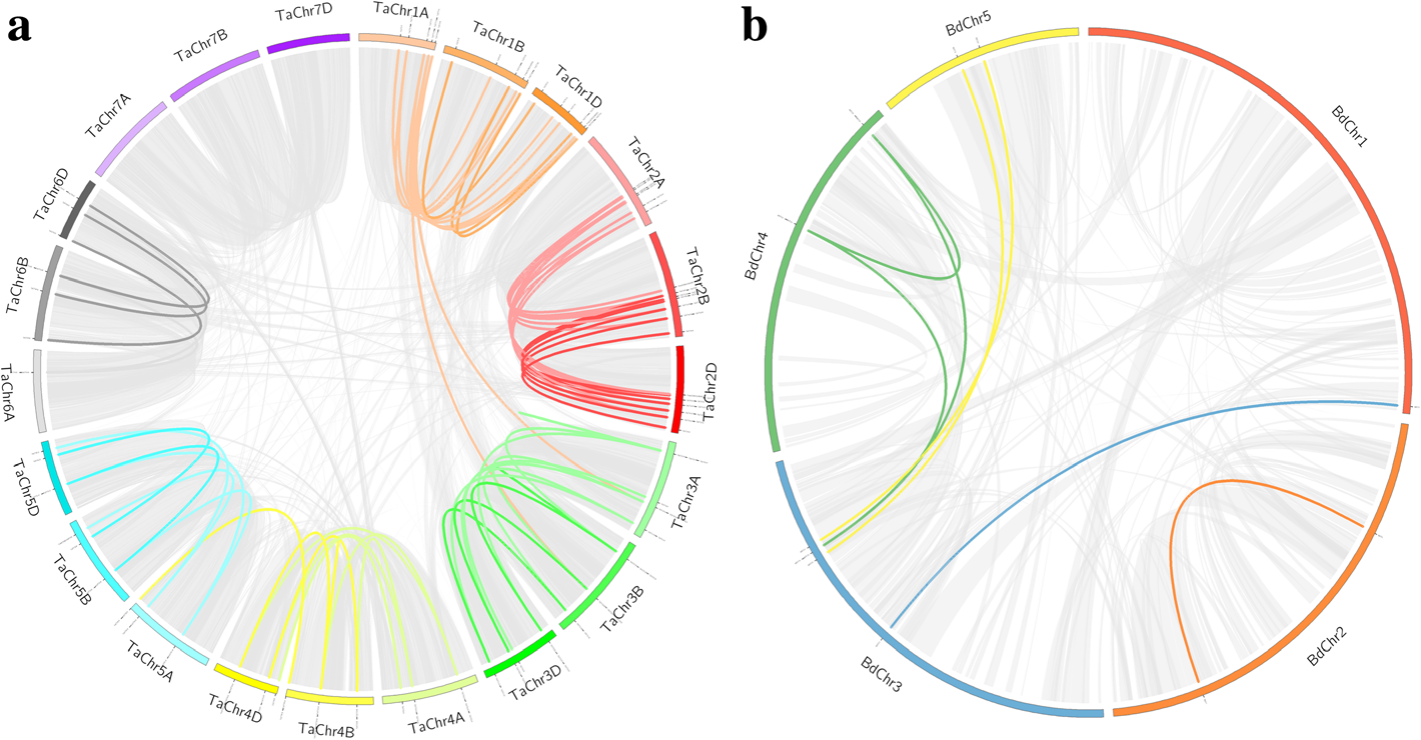
Schematic representations for the inter-chromosomal relationships of wheat and *B. distachyon* genes. **a** Duplicated Trihelix gene pairs in wheat. **b** Duplicated Trihelix gene pairs in *B. distachyon*

To identify orthologous of *TaGT* and *BdGT* genes, 67 and 7 pairs of putative paralogous of *TaGT* (Additional file 16; Fig. 5a) *BdGT* genes (Additional file 17; Fig. 5b) were identified. These results were consistent with phylogenetic analyses.

**Fig. 6:**
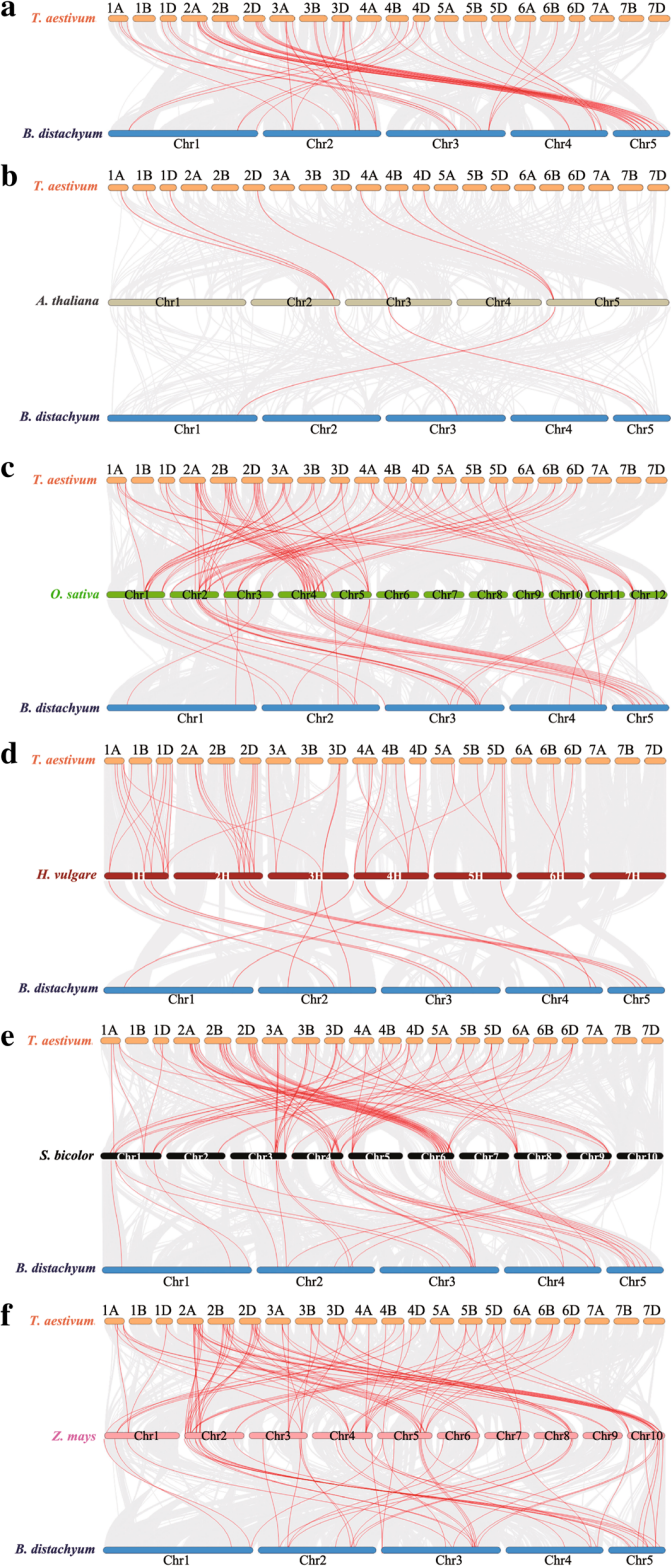
Syntenic relationships between *Trihelix* genes in different species. Gray lines in the background indicate the collinear blocks within wheat/*B. distachyon* and other plant genomes, while the red lines highlight the syntenic Trihelix gene pairs. **a** Orthologous relationship of *Trihrlix* genes between wheat and *B. distachyon*. Results of orthologous relationship analysis of Trihrlix genes between **b** wheat/*B. distachyon* and *Arabidopsis*, **c** wheat/*B. distachyon* and rice, **d** wheat/*B. distachyon* and *Sorghum bicolor* **e** wheat/*B. distachyon* and *Zea mays*, **f** wheat/*B. distachyon* and *Arabidopsis*. Comparative physical mapping shows the orthologous relationships of BdGT TFs with **g** *Oryza sativa*, **h** *Sorghum bicolor* and **i** *Zea mays*

In addition, orthologous between *Trihelix* genes in wheat, *B. distachyon, Arabidopsis thaliana,* rice, *Hordeum vulgare*, *Sorghum bicolor*, and *Zea mays* were also investigated. A total of 53, 7, 88, 39, 86 and 154 orthologous *Trihelix* gene pairs were found between wheat and *B. distachyon* (Additional file 18; Fig. 6a), *Arabidopsis thaliana* (Additional file 19; Fig. 6b), rice (Additional file 20; Fig. 6c), *Hordeum vulgare* (Additional file 21; Fig. 6d), *Sorghum bicolor* (Additional file 22; Fig. 6e), *Zea mays* (Additional file 23; Fig. 6f) respectively*.* Moreover, 3, 25, 14, 21 and 22 orthologous *Trihelix* gene pairs between *B. distachyon* and *Arabidopsis thaliana* (Additional file 24; Fig. 6b), rice (Additional file 25; Fig. 6c), *Hordeum vulgare* (Additional file 26:; Fig. 6d), *Sorghum bicolor* (Additional file 27; Fig. 6e), and *Zea mays* (Additional file 28; Fig. 6f) were also identified respectively. These results suggested that *Trihelix* genes of monocot have strong relationships.

The functional conservation of gene sets was further investigated by comparing both, *BdGT18* and *BdGT20* gene sets, in detail. A total of 65 and 54 *cis-*elements were found in *BdGT18* and *BdGT20*, respectively. Among them, 33 were common (Additional files 10 and 11). Furthermore, given that the expression pattern between both gene sets was very similar (Figs. 8 and 10). *BdGT18* and *BdGT20* may share similar functions.

To better understand the evolutionary factors that affect the *Trihelix* gene family, we calculated Ka and Ks ratio between of *Trihelix* gene pairs. The ratio of most of the tandem and segmental duplications TaGT and BdGT gene pairs, as well as of the orthologous Trihelix gene pairs was less than 1, suggesting that this gene family might have undergone strong purifying selective pressure during evolution in wheat and *B. distachyon* (Additional files 16, 17, 18, 19, 20, 21, 22, 23, 24, 25, 26, 27 and 28).

### Expression pattern analyses

To analyze expression pattern associated with gene function, we first investigated the temporal and spatial expression patterns of *Trihelix* genes in wheat and *B. distachyon*, based on public RNA-sequencing data (Additional files 29 and 30). In general, there was no obvious pattern.

**Fig. 7:**
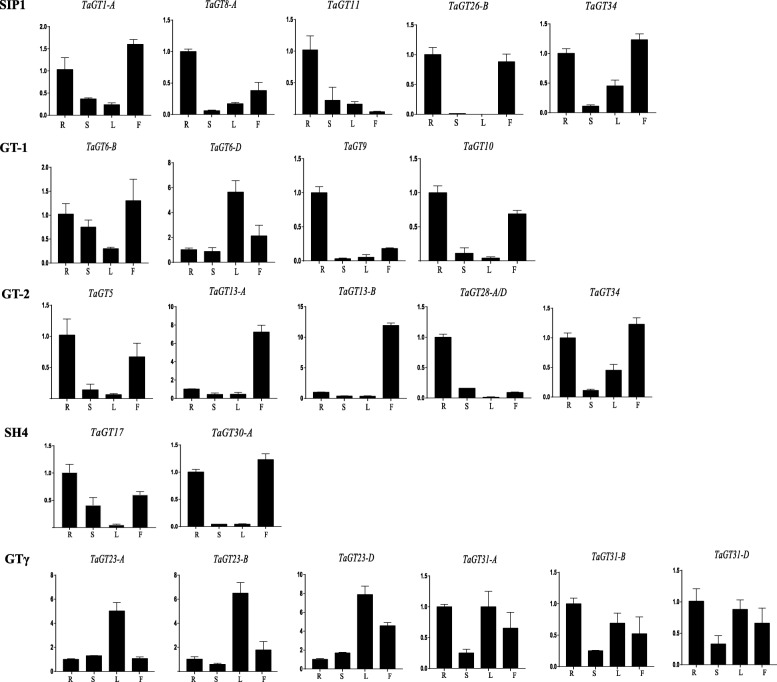
Expression profiles of *TaGTs* in different tissue (R, roots; S, stems; L, leaves; F, flowers)

**Fig. 8:**
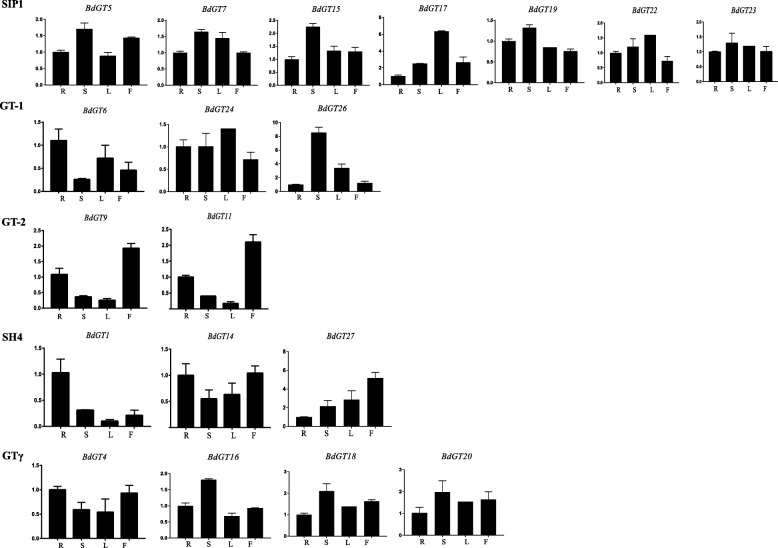
Expression profiles of *BdGTs* in different tissues (R, roots; S, stems; L, leaves; F, flower

**Fig. 9:**
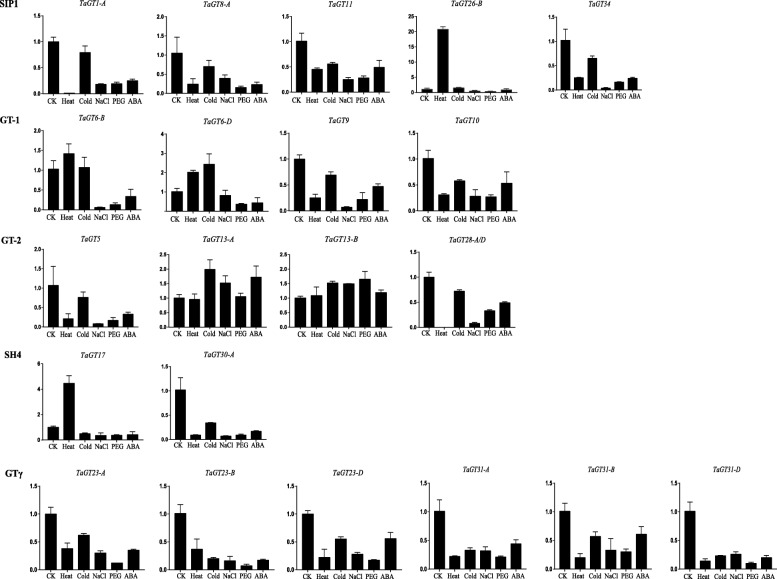
The expression level of *TaGTs* under different abiotic stresses

**Fig. 10:**
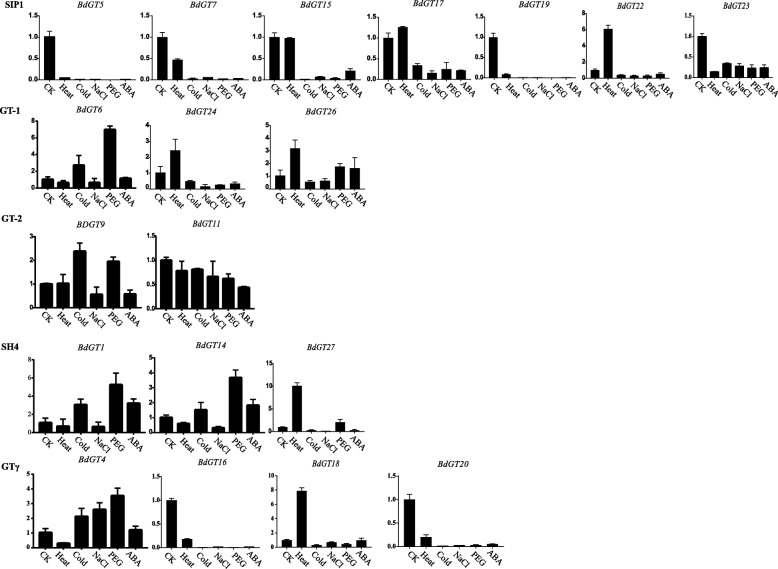
The expression level of *BdGTs* under different abiotic stresses

Furthermore, we performed qRT-PCR to analyze the expression patterns of 22 and 19 randomly selected *TaGT* and *BdGT* genes, respectively (Additional file 31). Briefly, clade members of GT-1, GT-2, SIP1 and SH4 were lowly expressed in stems and leaves, and highly expressed in roots and flowers (Fig. 7). GTγ genes were highly expressed in leaves. Different from the expression patterns of *TaGT* genes, the expression of BdSIP1 and BdGTγ genes was constitutive (Fig. 8). The expression of BdGT-2 and BdSH4 genes was similar to that of TaGT-2 and TaSH4 genes. The expression of different GT-1 genes showed relatively similarities. These results suggest that the functions of *BdGT* genes diversified and became more extensive during the evolutionary process. We also detected the expression of selected genes under different conditions of abiotic stress. Although, for most of the genes the expression was down-regulated under different abiotic stress conditions, for 11 genes (*TaGT6-B*, *TaGT6-D*, *TaGT13-B*, *TaGT17*, *TaGT26-B*, *BdGT17*, *BdGT18*, *BdGT22, BdGT24, BdGT26, BdGT27*) the expression was up-regulated under heat treatment (Figs. 9 and 10).

## Discussion

### Extensive functions of *Trihelix* genes

Previous studies demonstrated that *Trihelix* genes play extensive roles in plant growth/development and response to abiotic stress. In this study, the expression of most selected *BdGT* genes was constitutive, implying their multi-functions in growth and development. However, up- and down- regulated expression under different kinds of abiotic stress conditions (Figs 9 and 10) suggest that *Trihelix* genes also participate in the response to abiotic stress. GO analyses showed that *TaGT* and *BdGT* genes are grouped in 19 functional groups, including 10 involved in biological processes, 6 in cellular components and 3 in molecular functions, further indicating the extensive functions of *Trihelix* genes. Consistent with these results, many *cis*-elements were detected in promoters of *TaGT* and *BdGT* genes (Additional files 10 and 11). Most of the *cis*-elements were involved in plant growth/development and abiotic stress response.

Our results imply that the Trihelix gene family expanded during the evolutionary process. The number of genes increased and new subgroups emerged. Undoubtedly, the functions of *Trihelix* genes are shown to become more extensive with gene expansion.

Additionally, the functions of homologous genes might have diversified during the evolutionary process. For example, OsGTγ-1 is mainly expressed in leaves and functions in response to salt stress [18]. There are two homologous genes in wheat (*TaGT23* and *TaGT31*) and *B. distachyon* (*BdGT18* and *BdGT20*), respectively. Proteins with similarly motif composition were grouped together, despite presenting a different gene structure (*OsGTγ-1* has two exons; *TaGT23*, *TaGT31*, *BdGT18* and *BdGT20* only have one exon respectively) (Additional file 32).

Like *OsGTγ-1*, three copies of *TaGT-23* and *TaGT-31* were mainly expressed in leaves. Consistently, *TaGT-23* and *TaGT-31* share 62 and 64 common cis-elements with rice, respectively. But when treated with salt stress, the expression of *TaGT-23* and *TaGT31* was down-regulated, while the expression of *OsGTγ-1* was up-regulated. Different from *OsGTγ-1*, the expression of *BdGT18* and *BdGT20* was constitutive and they were expressed in roots, stems, leaves and flowers at high levels, although they shared 53 and 35 common cis-elements with *OsGTγ-1*, respectively. However, the expression of *BdGT18* and *BdGT20* was also down-regulated under salt stress. Such different expression patterns indicate diversified functions.

### Conservation and diversification between *TaGT* and *BdGT* genes

Gene structure of *TaGT* and *BdGT* genes was similar within the same subfamilies (Figs. 3 and 4). In the promoters of 80 *TaGT* genes, 249 kinds of *cis*-elements were detected (Additional file 10); in the promoters of 27 *BdGT* genes, 233 kinds of *cis*-elements were detected (Additional file 11). Among these *cis*-elements, 216 are the same. A total of 10 protein motifs were found. Among them, motif 1 and 3 were found in most TaGT and BdGT proteins; motif 7 was present in GTγ members, while motif 10 was only found in the SH4 subgroup (Figs. 3 and 4). These results are consistent with the fact that wheat and *B. distachyon* have a close phylogenetic relationship. Furthermore, GO annotations showed that TaGT and BdGT proteins were divided into 19 functional groups (Additional file 15). Taken these results together, it is possible to conclude that *TaGT* and *BdGT* genes are conserved to some extent.

On the other hand, the expression pattern of TaGT genes is quite different from that of BdGT genes. Most *TaGT* genes are mainly expressed in roots and flowers, while most *BdGT* genes are expressed constitutively (Figs. 7 and 8). During the evolutionary process, the expression domain of *BdGT* genes might have extended. As a result, the functions of single *BdGT* gene might have been more extensive. Furthermore, up- and down- regulated expression of *TaGTs* and *BdGTs* under different kinds of abiotic stresses (Figs. 9 and 10), suggest that *Trihelix* genes participate in plant response to abiotic stress.

## Conclusion

In this paper, the *Trihelix* genes were analyzed at the genome level in wheat and *B. distachyon*. 80 *TaGT* and 27 *BdGT* genes were identified. Gene structure, protein motifs, GO analyses, the *cis*-elements and the expression pattern indicate the conservative and diversified nature of *TaGT* and *BdGT* genes.

## Methods

### Identification and analyses of TaGT and BdGT TFs

To identify the Trihelix TFs in 51 species, firstly, we used the Hidden Markov Model profile (PF13837) of the Trihelix signature domain downloaded from the Pfam v31.0 database (http://pfam.xfam.org/) to search against plant protein sequences using a threshold of E < 1e− 5 [30]. Then, 31 rice and 30 *Arabidopsis* Trihelix protein sequences were selected to search against protein sequences using a threshold of E < 1e− 5 and an identity of 50%. Subsequently, blast and manual corrections were performed to remove alternative events and redundancy. Finally, the NCBI-CDD web server [31] and SMART [32] were used to confirm whether putative Trihelix TFs had conserved GT domains. To further verify the existence of *Trihelix* genes in wheat and *B. distachyon*, we performed BLASTN to search for EST using putative *Trihelix* gene sequences. The MapInspect tool (http://mapinspect.software.informer.com/) was used to map the chromosome location. The ProtParam tool was used to predict physical and chemical properties of TaGT and BdGT TFs [33].

### Multiple sequence alignment and phylogenetic analyses

To investigate the genetic divergence between each group, we counted the genetic distances based on amino acid sequences using the method of Min Jiang et al. [34]. Full-length Trihelix protein sequences were aligned using the T-COFFEE method [35]. NJ and ML trees were constructed with 1000 bootstrap replicates using MEGA 7 EvolView for tree visualization (http://www.evolgenius.info/evolview/).

### Gene structure and conserved motif analyses

Gene structures were deduced using GSDS (http://gsds.cbi.pku.edu.cn/). The MEME Suite web server (http://meme-suite.org/) was used to predict the conserved motifs of TaGT and BdGT TFs, with the maximum number and optimum width of motif sets at 10 and 5 to 200 amino acids, respectively [36].

### Promoter analysis of GT genes and gene ontology (GO) annotation

The 1500-bp upstream genomic DNA sequences of the wheat and *B. distachyon Trihelix* genes were submitted to PLANT CARE database (http://bioinformatics.psb.ugent.be/webtools/plantcare/html/)to predict the *cis*-acting elements [37]. Gene ontology (GO) annotation of Trihelix proteins was obtained from the PLAZLA3.0 (https://bioinformatics.psb.ugent.be/plaza/versions/plaza_v3_monocots/genome_mapping) to predict the functions of TaGT and BdGT proteins. GO annotation were then plotted using the OmicShare tool (http://www.omicshare.com/tools).

### Detection of homologous gene pairs and synteny analysis

Gene duplication analysis of Trihelix genes in different species was performed using the Multiple Collinearity Scan toolkit (MCScanX) with default parameters [38]. The collinear chart of *TaGT* and *BdGT* genes was drawn using Circos v0.55 [39]. We plotted the synteny relationship of the *Trihelix* genes from selected species and calculated the ratio of non-synonymous (ka) to synonymous (ks). Substitutions (Ka/Ks) using the TBtools software (https://github.com/CJ-Chen/TBtools) [40].

### Plant materials and qRT-PCR

The wheat cultivar ‘Chinese Spring’ and *B. distachyon* Bd-21 were planted in a growth chamber at 23 ± 1 °C with a photoperiod of 16 h /8 h (light/dark). Roots, stems, leaves, and inflorescences were collected at the heading stage. The methods used for abiotic stress treatment, primer design, RNA extraction, cDNA synthesis, quantitative RT-PCR were described in a previous study [26]. To explore the expression profiles of GT genes in different tissues and abiotic stress, the FPKM values of expansin genes were obtained from ArrayExpress database under accession number E-MTAB-4401 and E-MTAB-4484, the RNA-seq dataset in the SRA database with accession number SRP045409 and PRJNA360513.

## Additional files

Additional file 1: The detailed information of *TaGT* genes. (XLSX 22 kb)

Additional file 2: The detailed information of *BdGT* genes. (XLSX 16 kb)

Additional file 3: Chromosome location of wheat *Trihelix* genes. (PDF 376 kb)

Additional file 4: Chromosome location of *B. distachyon Trihelix* genes. (PDF 100 kb)

Additional file 5: Multiple sequence alignment of GT domains of wheat and *B. distachyon* Trihelix TFs. Helix 1, 2 and 3 rectangular bars represent trihelix structure identified in the GT domain. Yellow shade indicates conserved amino acids. (PDF 6136 kb)

Additional file 6: Conserved amino acids and numbers in the Helix1, 2, 3 which were from the GT domain of *TaGTs*, *BdGTs* and *OsGTs*. (XLSX 12 kb)

Additional file 7: Representing number of *Trihelix* genes present in different plant species. (XLSX 15 kb)

Additional file 8: The 31 *Oryza sativa Trihelix* genes, 50 *Zea mays Trihelix* genes, 25 *Hordeum vulgare Trihelix* genes and 30 *Sorghum bicolor Trihelix* genes. (XLSX 16 kb)

Additional file 9: Phylogenetic tree of Trihelix proteins by NJ. The five different subfamilies were indicated by different colors. Trihelix proteins of TaGT and BdGT TFs were indicated by red and blue circles respectively. (PDF 595 kb)

Additional file 10: The *cis*-elements in promoter sequences of *Trihelix* genes in wheat. (XLSX 89 kb)

Additional file 11: The *cis*-elements in promoter sequences of *Trihelix* genes in *B. distachyon*. (XLSX 41 kb)

Additional file 12: The promoter regions of *TaGT* and *BdGT* genes contained plant growth/development - and stress response-related *cis*-elements. (XLSX 29 kb)

Additional file 13: More than 80% of *TaGTs* and *BdGTs* contained plant growth/development - and stress response-related *cis*-elements. (XLSX 20 kb)

Additional file 14: The promoter regions of *TaGT* and *BdGT* genes in different subfamilies. (XLSX 14 kb)

Additional file 15: Functional categorization of *Trihelix* genes in wheat and *B. distachyon*. *TaGT* and *BdGT* genes were categorized according to Gene Ontology annotation. The number and proportion of each category were displayed based on three functional classification categories (biological process, molecular function and cellular component). (PDF 284 kb)

Additional file 16: Paralogous TaGT gene pairs. (XLSX 18 kb)

Additional file 17: Paralogous BdGT gene pairs. (XLSX 13 kb)

Additional file 18: Orthologous Trihelix gene pairs between wheat and *B. distachyon*. (XLSX 17 kb)

Additional file 19: Orthologous Trihelix gene pairs between wheat and *Arabidopsis thaliana*. (XLSX 14 kb)

Additional file 20: Orthologous Trihelix gene pairs between wheat and *O.sativa*. (XLSX 19 kb)

Additional file 21: Orthologous Trihelix gene pairs between wheat and *Hordeum vulgare*. (XLSX 15 kb)

Additional file 22: Orthologous Trihelix gene pairs between wheat and *Sorghum bicolor*. (XLSX 18 kb)

Additional file 23: Orthologous Trihelix gene pairs between wheat and *Zea mays*. (XLSX 17 kb)

Additional file 24: Orthologous Trihelix gene pairs between *B. distachyon* and *Arabidopsis thaliana*. (XLSX 13 kb)

Additional file 25: Orthologous Trihelix gene pairs between *B. distachyon* and *O.sativa*. (XLSX 14 kb)

Additional file 26: Orthologous Trihelix gene pairs between *B. distachyon* and *Hordeum vulgare*. (XLSX 14 kb)

Additional file 27: Orthologous Trihelix gene pairs between *B. distachyon* and *Sorghum bicolor*. (XLSX 14 kb)

Additional file 28: Orthologous Trihelix gene pairs between *B. distachyon* and *Zea mays*. (XLSX 14 kb)

Additional file 29: Expression patterns of *TaGT* genes in various wheat tissues (a) and stresses (b).The expression data was collected through pubic RNA-seq data. The tissues expression of *TaGT* genes at different growth stages, such as roots (cotyledon emergence stage), stems (FL.02 1/2 of flowers open stage) leaves tissues (cotyledon emergence stage) and flowers (FL.02 1/2 of flowers open stage). Heatmap of expression profiles for *TaGT* genes across different stresses under 1 and 6 h’s treatments, including heat stress, drought stress and drought&heat combined stress. (PDF 461 kb)

Additional file 30: Expression patterns of *BdGT* genes in various B. *distachyon* (a) organs (Leaf, inflorescence, anther, pistil, plant embryo, and endosperm) and abiotic stresses (CK, ABA, SA, JA, PHX, and Ethylene). (PDF 364 kb)

Additional file 31: Gene-specific primer sequences for qRT-PCR. (XLSX 14 kb)

Additional file 32: Gene structure and motifs of .OsGTγ-1 protein. In the gene structure part, blue boxes represent UTRs, yellow boxes represent exons, and black lines represent introns. In the motif part, the boxes in different color represent different motifs, and the black lines represent un-conserved sequences. (PDF 254 kb)

Additional file 33: Raw data (including DNA sequences and amino acids) of *TaGT* and *BdGT* genes sequences. (FA 396 kb)

## Abbreviations

EST: Expressed Sequence Tag
GO: Gene Ontology
GRAVY: Grand average of hydropathy
Ka: Non-synonymous
Ks: Synonymous
ML: Maximum Likelihood
NJ: Neighbor-joining
PI: Isoelectric Point
TFs: Transcription factors

## References

[1] Gourrierec J, Le E (1999). Transcriptional activation by Arabidopsis GT-1 may be through interaction with TFIIA–TBP–TATA complex. Plant J.

[2] Green PJ, Kay SA, Chua NH (1987). Sequence-specific interactions of a pea nuclear factor with light-responsive elements upstream of the rbcS-3A gene. EMBO J.

[3] Kaplan-Levy RN, Brewer PB, Quon T, Smyth DR (2012). The trihelix family of transcription factors--light, stress and development. Trends Plant Sci.

[4] Ji J, Zhou Y, Wu H, Yang L (2015). Genome-wide analysis and functional prediction of the Trihelix transcription factor family in rice. Hereditas.

[5] Wang W, Wu P, Liu TK, Ren H, Li Y, Hou X (2017). Genome-wide Analysis and Expression Divergence of the Trihelix family in *Brassica Rapa* : Insight into the Evolutionary Patterns in Plants. Sci Rep.

[6] Yu C, Cai X, Ye Z, Li H (2015). Genome-wide identification and expression profiling analysis of trihelix gene family in tomato. Biochemi Bioph Res Co.

[7] Song A, Gao T, Wu D, Xin J, Chen S, Guan Z, Wang H, Jin L, Chen F (2016). Transcriptome-wide identification and expression analysis of chrysanthemum SBP-like transcription factors. Int J of Mol Sci.

[8] Wang XH, Li QT, Chen HW, Zhang WK, Ma B, Chen SY, Zhang JS (2014). Trihelix transcription factor GT-4 mediates salt tolerance via interaction with TEM2 in Arabidopsis. BMC Plant Biol.

[9] Yu C, Song L, Song J, Ouyang B, Guo L, Shang L, Wang T, Li H, Zhang J, Ye Z (2018). ShCIGT, a Trihelix family gene, mediates cold and drought tolerance by interacting with SnRK1 in tomato. Plant Sci.

[10] O’Brien M, Kaplanlevy RN, Quon T, Sappl PG, Smyth DR (2015). PETAL LOSS, a trihelix transcription factor that represses growth in Arabidopsis thaliana, binds the energy-sensing SnRK1 kinase AKIN10. J Exp Bot.

[11] Gao MJ, Lydiate DJ, Xiang L, Lui H, Gjetvaj B, Hegedus DD, Rozwadowski K (2009). Repression of seed maturation genes by a trihelix transcriptional repressor in Arabidopsis seedlings. Plant Cell.

[12] Xi J, Qiu Y, Du L, Poovaiah BW (2012). Plant-specific trihelix transcription factor AtGT2L interacts with calcium/calmodulin and responds to cold and salt stresses. Plant Sci.

[13] Zheng X, Liu H, Ji H, Wang Y, Dong B, Qiao Y, Liu M, Li X (2016). The wheat GT factor TaGT2L1D negatively regulates drought tolerance and plant development. Sci Rep.

[14] Fan M, Herburger K, Jensen JK, Zemelis-Durfee S, Brandizzi F, Fry SC, Wilkerson CG (2018). A Trihelix family transcription factor is associated with key genes in mixed-linkage glucan accumulation. Plant Physiol.

[15] Kitakura S, Fujita T, Ueno Y, Terakura S, Wabiko H, Machida Y (2002). The protein encoded by oncogene 6b from agrobacterium tumefaciens interacts with a nuclear protein of tobacco. Plant Cell.

[16] Ming-Jun G, Xiang L, Helen L, Gropp GM, Lydiate DD, Shu W, Hegedus DD (2011). ASIL1 is required for proper timing of seed filling in Arabidopsis. Plant Signal Behav.

[17] Luo J, Tang S, Mei F, Peng X, Li J, Li X, Yan X, Zeng X, Liu F, Wu Y (2017). BnSIP1–1, a Trihelix Family Gene, Mediates Abiotic Stress Tolerance and ABA Signaling in *Brassica napus*. Frontiers Plant Sci.

[18] Fang Y, Xie K, Hou X, Hu H, Xiong L (2010). Systematic analysis of GT factor family of rice reveals a novel subfamily involved in stress responses. Mol Genet Genomics.

[19] Liujun D, Mingyu P, Bo W, Xianping W, Renchun F, Xiangqi Z (2016). Transcription factor gene TuGTγ-3 is involved in the stripe rust resistance in Triticum urartu. Yi Chuan.

[20] Bo L, Shan J, Xiao Y, Cheng C, Sixue C, Yanbing C, Yuan JS, Daohong J, Ping H, Libo S (2015). Phosphorylation of trihelix transcriptional repressor ASR3 by MAP KINASE4 negatively regulates Arabidopsis immunity. Plant Cell.

[21] Zhongwei L, Megan EG, Xianran L, Zuofeng Z, Lubing T, Yongcai F, Wenxu Z, Xiangkun W, Daoxin X, Chuanqing S (2007). Origin of seed shattering in rice (Oryza sativa L.). Planta.

[22] Yue L, Xiaodong L, Yongmei D, Zongming X, Shouyi C (2015). Cloning and functional analysis of the cotton Trihelix transcription factor GhGT29. Yi chuan.

[23] Wusirika R, Jorge D, Yong-Jin P, Carlos B, John E, Phillip SM, Bennetzen JL. Different types and rates of genome evolution detected by comparative sequence analysis of orthologous segments from four cereal genomes. Genetics. 2002;162(3):1389. 10.1093/genetics/162.3.1389 PMC1462341 12454082

[24] Appels R, Eversole K, Feuillet C, Keller B, Rogers J, Stein N, Pozniak CJ, Stein N, Choulet F, Distelfeld A (2018). Shifting the limits in wheat research and breeding using a fully annotated reference genome. Science.

[25] Vogel JP, Garvin DF, Mockler TC, Schmutz J, Rokhsar D, Bevan MW, Barry K, Lucas S, Harmon-Smith M, Lail K (2010). Genome sequencing and analysis of the model grass Brachypodium distachyon. Nature.

[26] Chen S, Niu X, Guan Y, Li H. Genome-wide analysis and expression profile of the MYB genes in Brachypodium distachyon. Plant Cell Physiol. 2017;58(10):1777-1788. 10.1093/pcp/pcx115 29016897

[27] Kay SA, Chua NH. The rice phytochrome gene: structure, autoregulated 707 expression, and binding of GT-1 to a conserved site in the 5′ upstream 708 region. Plant Cell. 1989;1(3):351. 10.1105/tpc.1.3.351 PMC159767 2535506

[28] Todeschini AL, Georges A, Veitia RA (2014). Transcription factors: specific DNA binding and specific gene regulation. Trends Gene.

[29] Martin C, Ellis N, Rook F (2010). Do transcription factors play special roles in adaptive variation?. Plant Physiol.

[30] Finn RD, Coggill P, Eberhardt RY, Eddy SR (2016). The Pfam protein families database: towards a more sustainable future. Nucleic Acid Res.

[31] Marchlerbauer A, Derbyshire MK, Gonzales NR, Lu S, Chitsaz F, Geer LY, Geer RC, He J, Gwadz M, Hurwitz DI (2015). CDD: NCBI's conserved domain database. Nucleic Acid Res.

[32] Letunic I, Doerks T, Bork P (2015). SMART: recent updates, new developments and status in 2015. Nucleic Acids Res.

[33] Wilkins MR, Gasteiger E, Bairoch A, Sanchez JC, Williams KL, Appel RD, Hochstrasser DF (1999). Protein identification and analysis tools in the ExPASy server. Methods Mol Biol.

[34] Jiang M, Chu Z (2018). Comparative analysis of plant MKK gene family reveals novel expansion mechanism of the members and sheds new light on functional conservation. BMC Genomics.

[35] Magis C, Taly JF, Bussotti G, Chang JM, Di TP, Erb I, Espinosacarrasco J, Notredame C (2013). T-Coffee: tree-based consistency objective function for alignment evaluation. Methods Mol Biol.

[36] Bailey TL, Elkan C (1994). Fitting a mixture model by expectation maximization to discover motifs in biopolymers. Proc Int Conf Intell Syst Mol Biol.

[37] Lescot M, Déhais P, Thijs G, Marchal K, Moreau Y, Peer YVD, Rouzé P, Rombauts S (2002). PlantCARE, a database of plant cis-acting regulatory elements and a portal to tools for in silico analysis of promoter sequences. Nucleic Acids Res.

[38] Yupeng W, Haibao T, Jeremy DD, Xu T, Jingping L, Xiyin W, Tae-ho L, Huizhe J, Barry M, Hui G (2012). MCScanX: a toolkit for detection and evolutionary analysis of gene synteny and collinearity. Nucleic Acids Res.

[39] Krzywinski M, Schein JI (2009). Circos: an information aesthetic for comparative genomics. Genome Res.

[40] Liu C, Xie T, Chen C, Luan A, Long J, Li C, Ding Y, He Y (2017). Genome-wide organization and expression profiling of the R2R3-MYB transcription factor family in pineapple ( Ananas comosus ). BMC Genomics.

