# Supplementary figures and images for: Comparison of Trihelix transcription factors between wheat and Brachypodium distachyon at genome-wide
Source: BMC Genomics. 2019 Feb 15;20:142. doi: 10.1186/s12864-019-5494-7 (PMC6377786; doi:10.1186/s12864-019-5494-7)

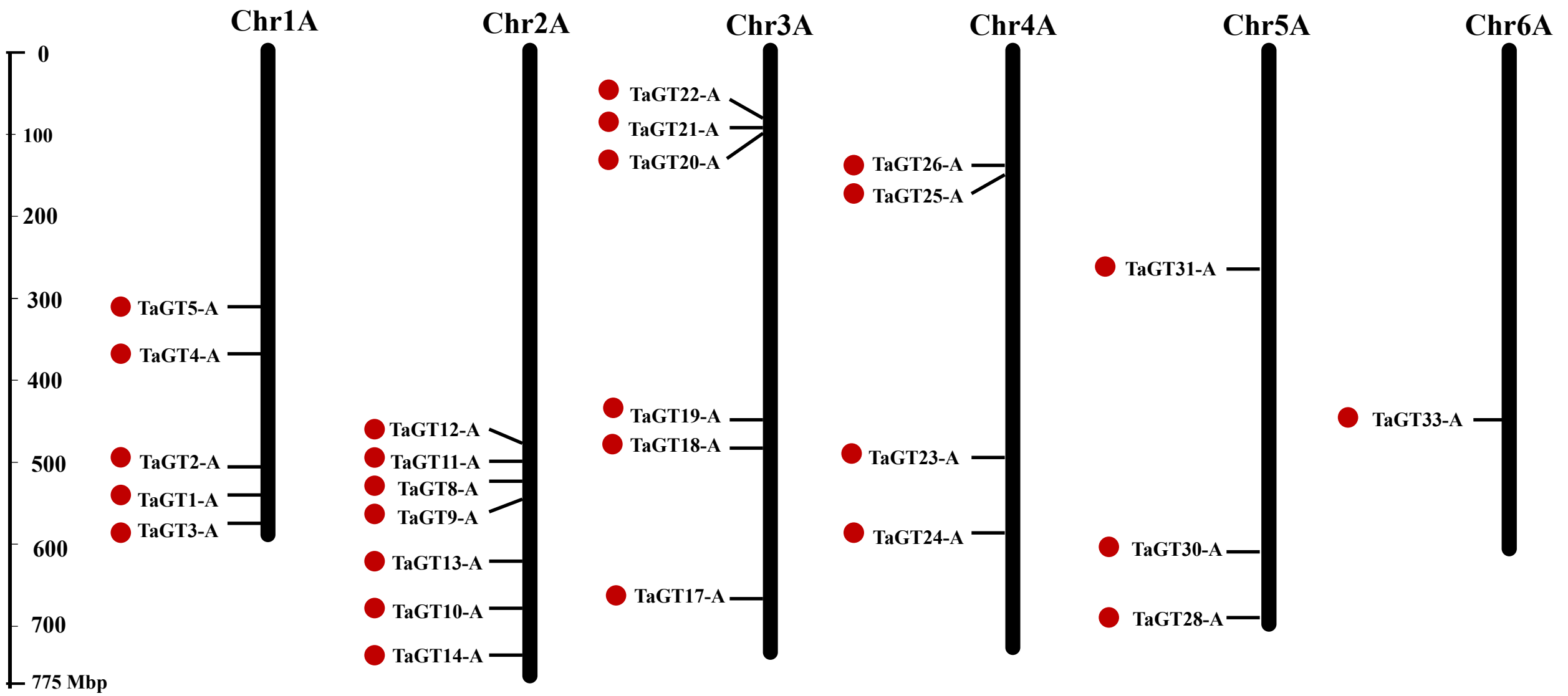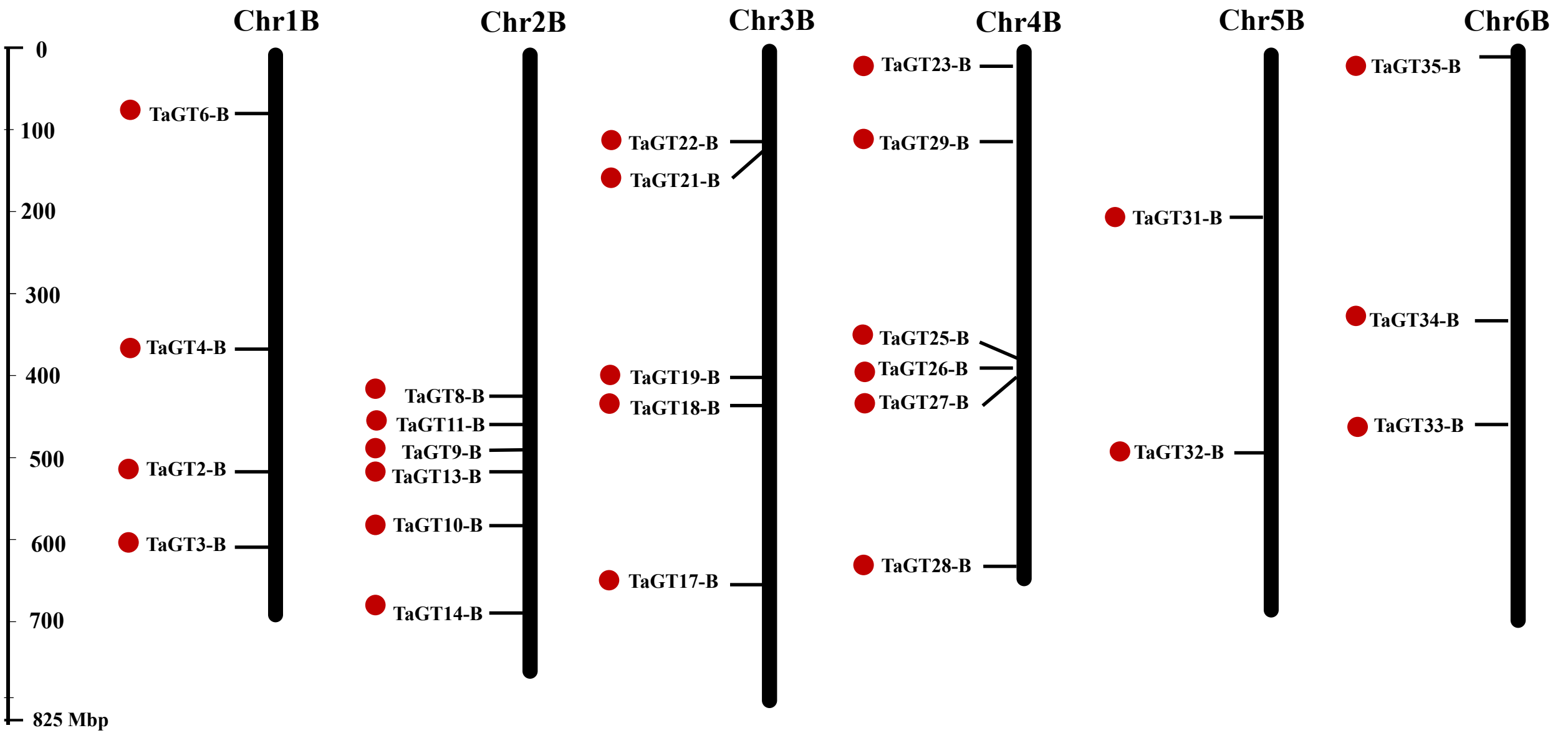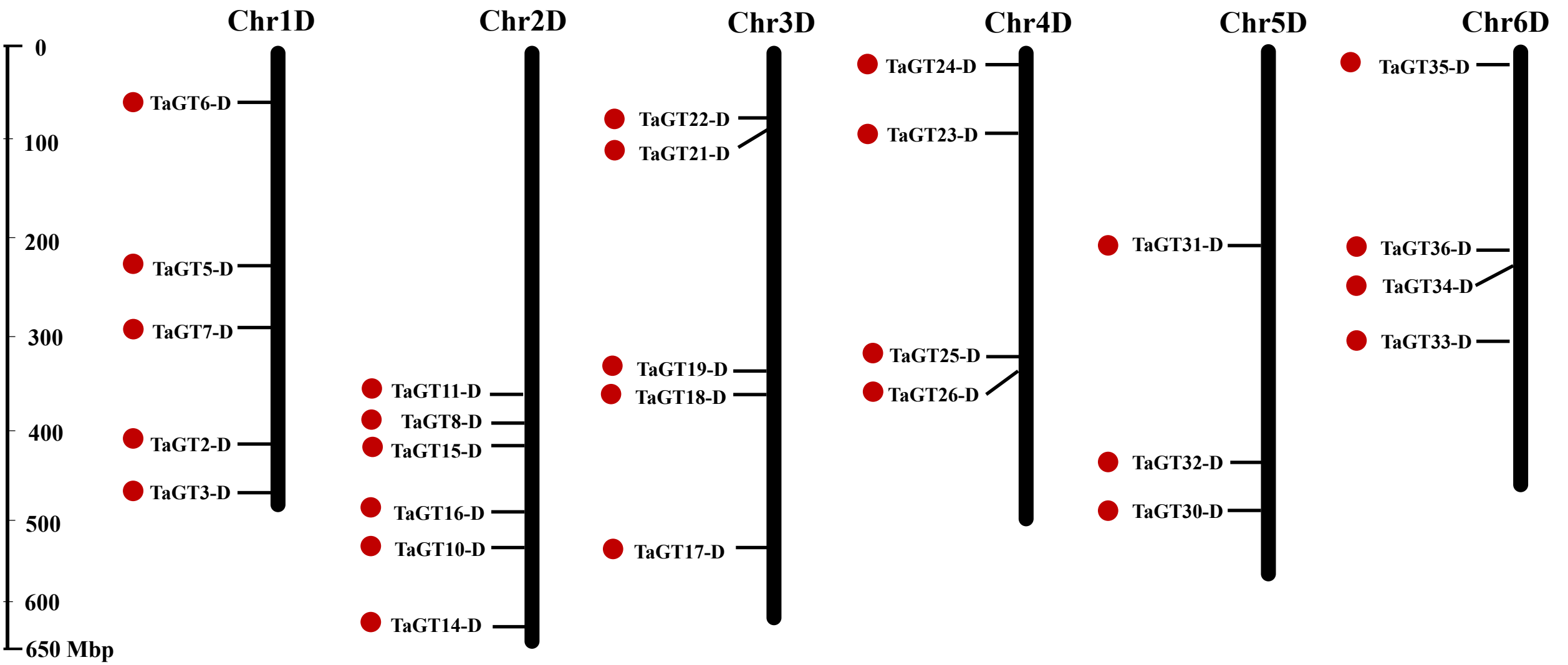

Supplement: Supplementary file 3 — Chromosome location of wheat Trihelix genes. (PDF 376 kb) [file 12864_2019_5494_MOESM3_ESM.pdf]

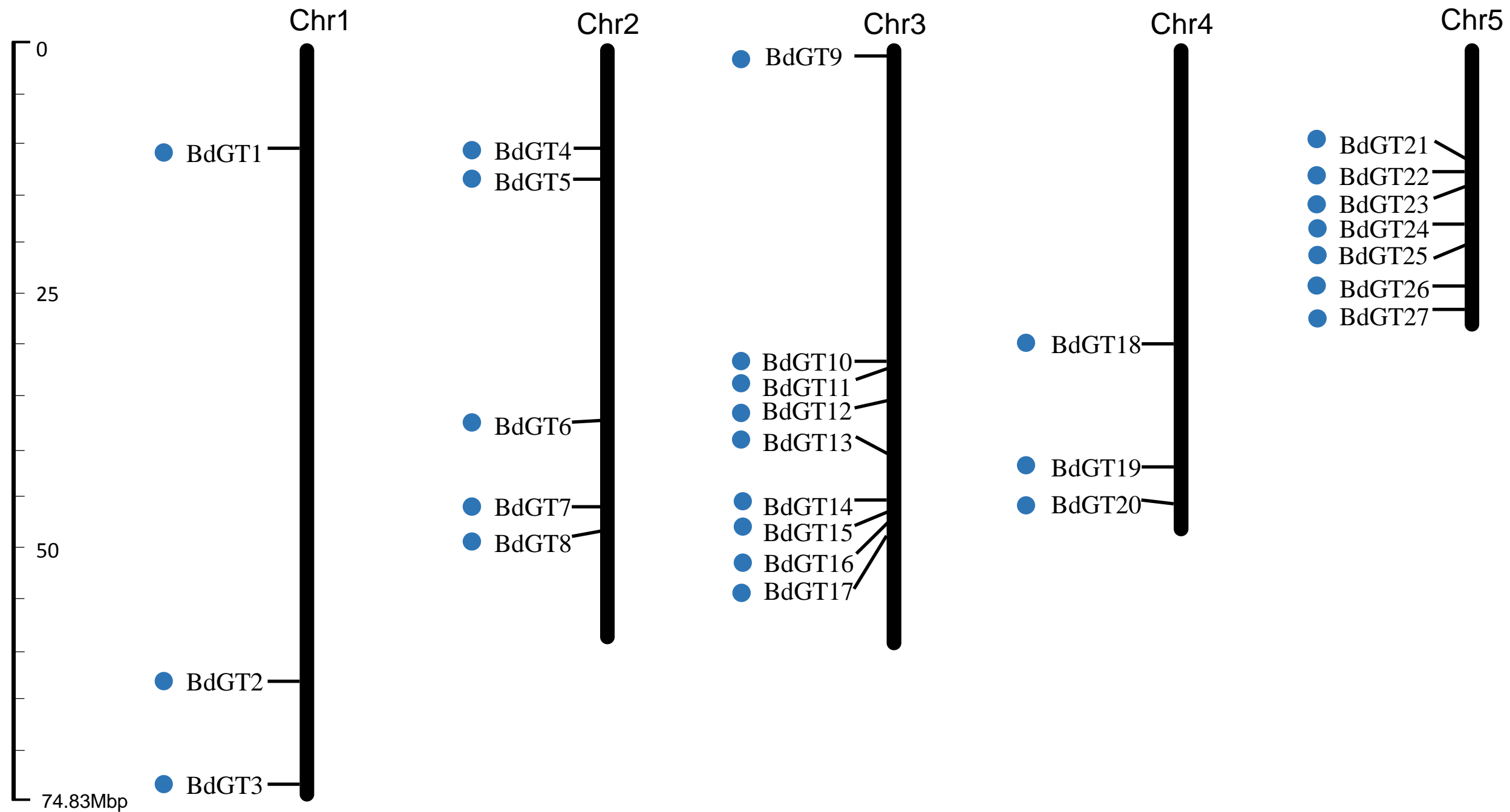

Supplement: Supplementary file 4 — Chromosome location of B. distachyon Trihelix genes. (PDF 100 kb) [file 12864_2019_5494_MOESM4_ESM.pdf]

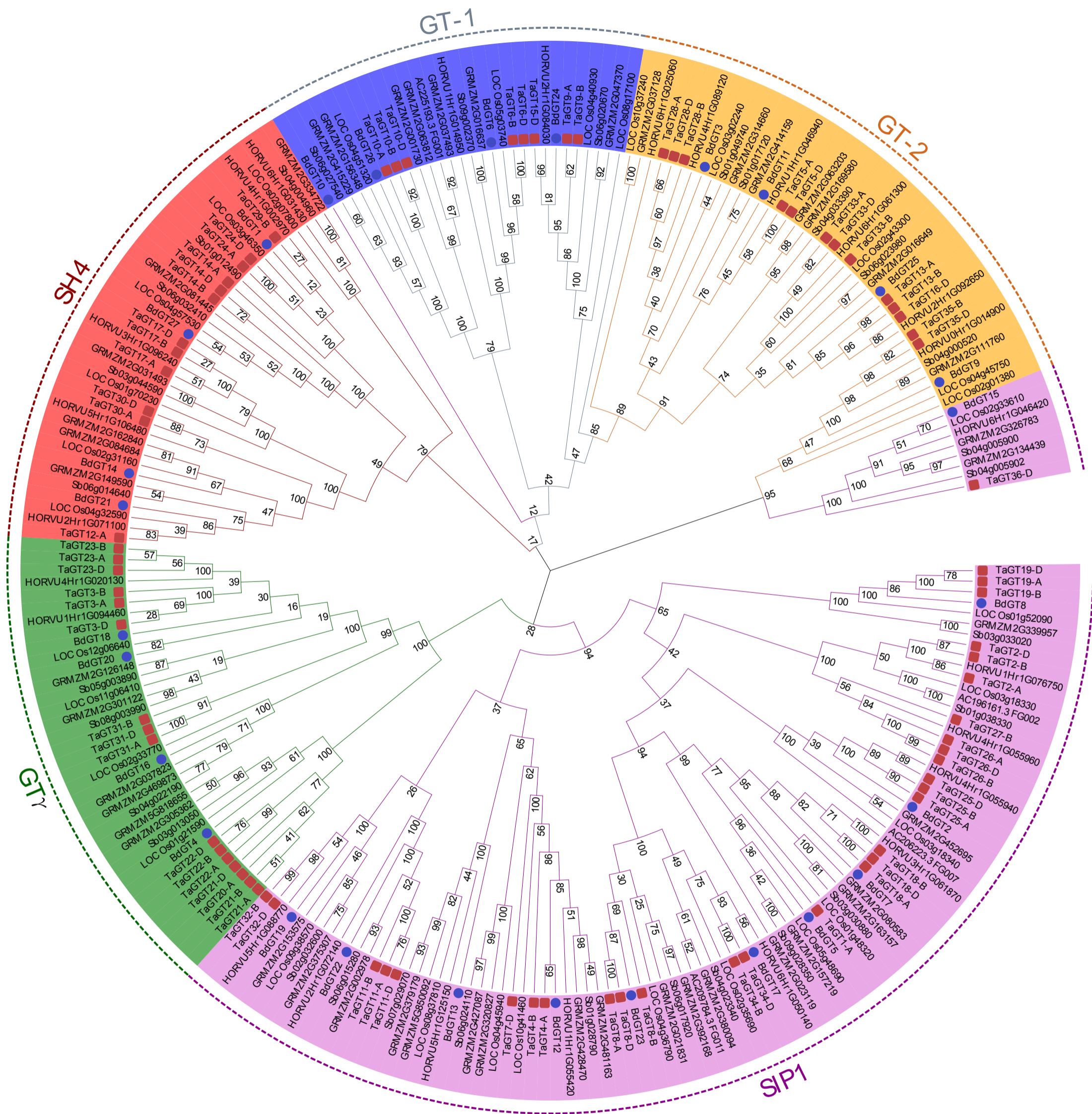

Supplement: Supplementary file 9 — Phylogenetic tree of Trihelix proteins by NJ. The five different subfamilies were indicated by different colors. Trihelix proteins of TaGT and BdGT TFs were indicated by red and blue circles respectively. (PDF 595 kb) [file 12864_2019_5494_MOESM9_ESM.pdf]

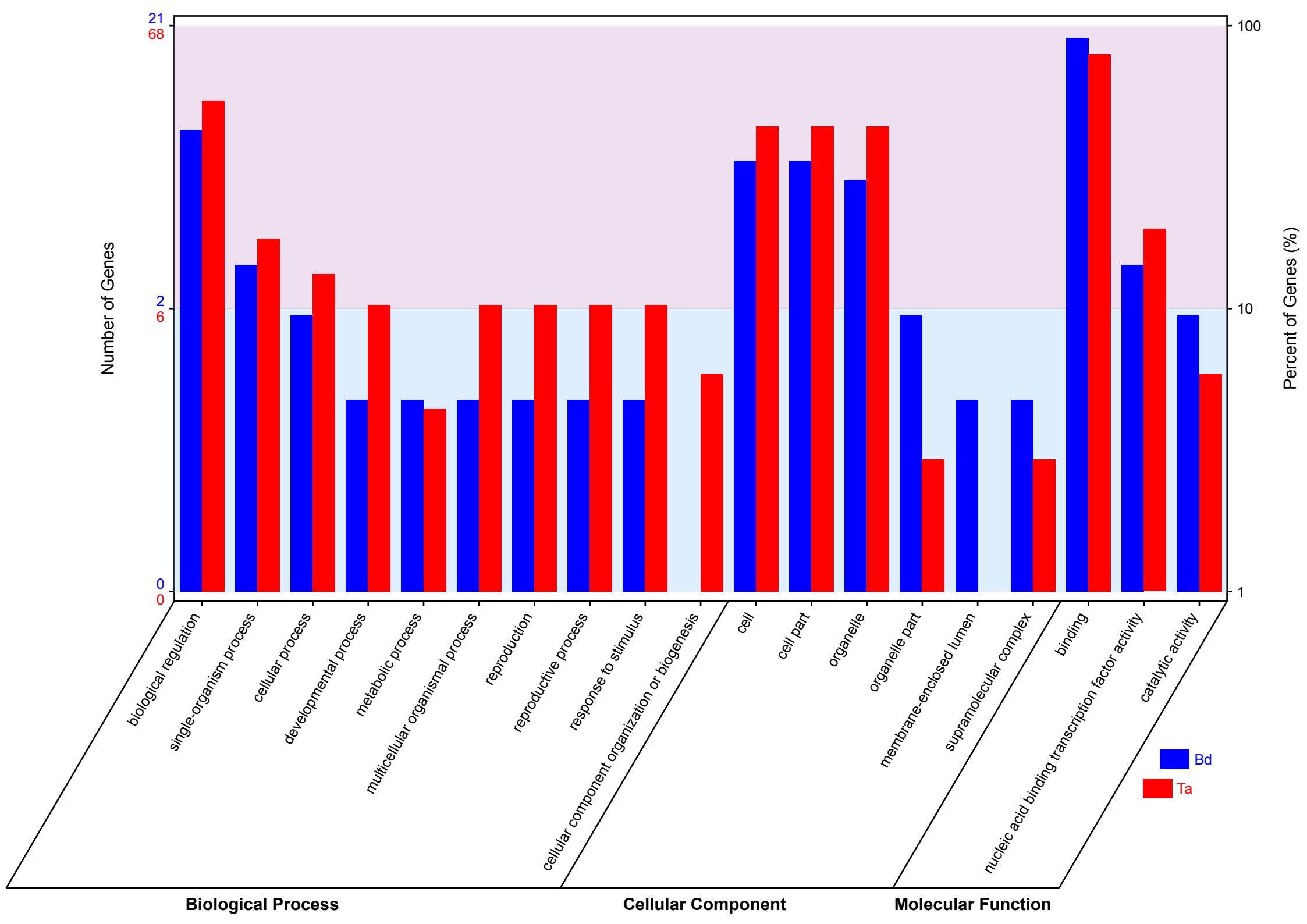

Supplement: Supplementary file 15 — Functional categorization of Trihelix genes in wheat and B. distachyon. TaGT and BdGT genes were categorized according to Gene Ontology annotation. The number and proportion of each category were displayed based on three functional classification categories (biological process, molecular function and cellular component). (PDF 284 kb) [file 12864_2019_5494_MOESM15_ESM.pdf]

a

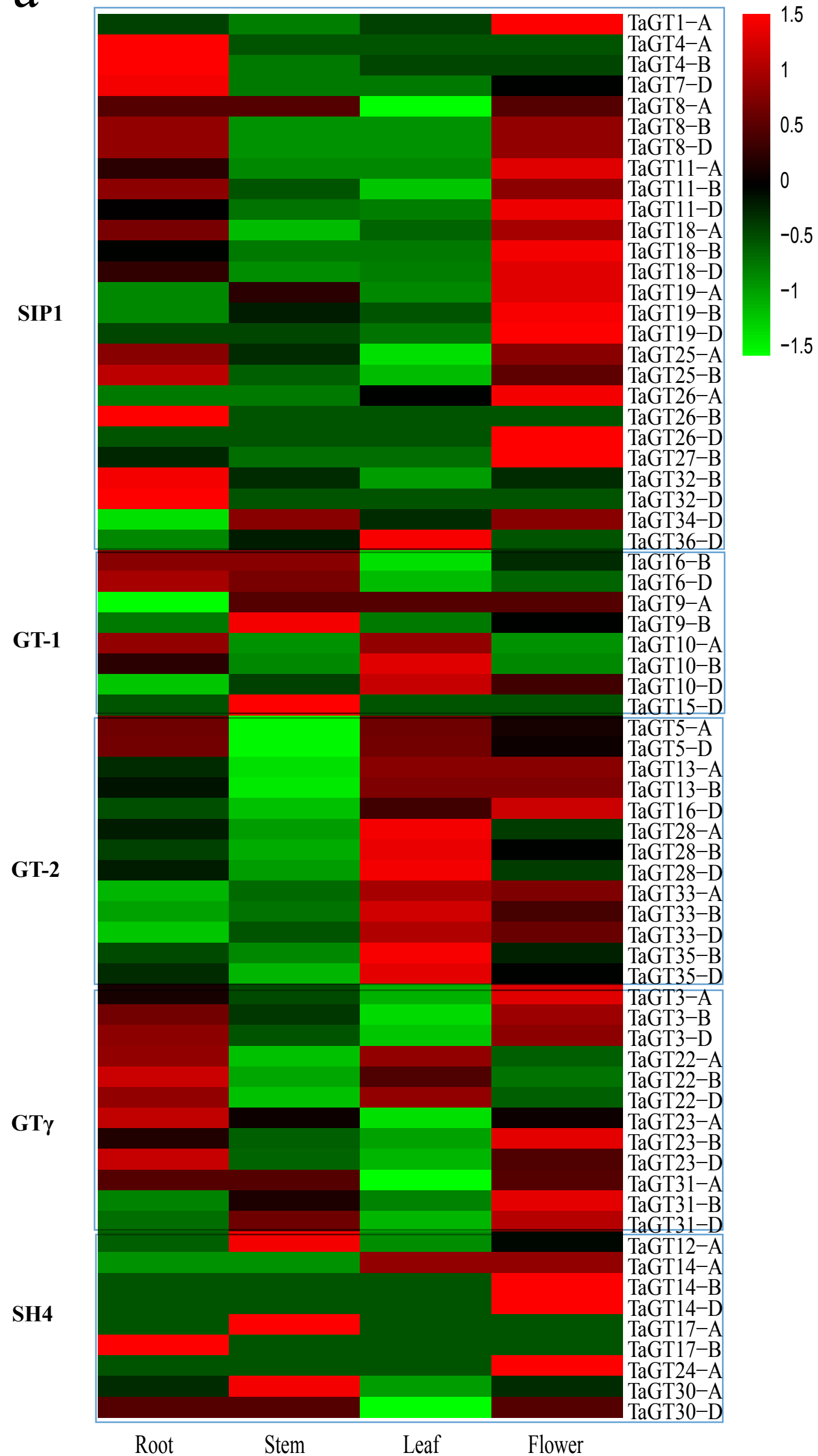

b

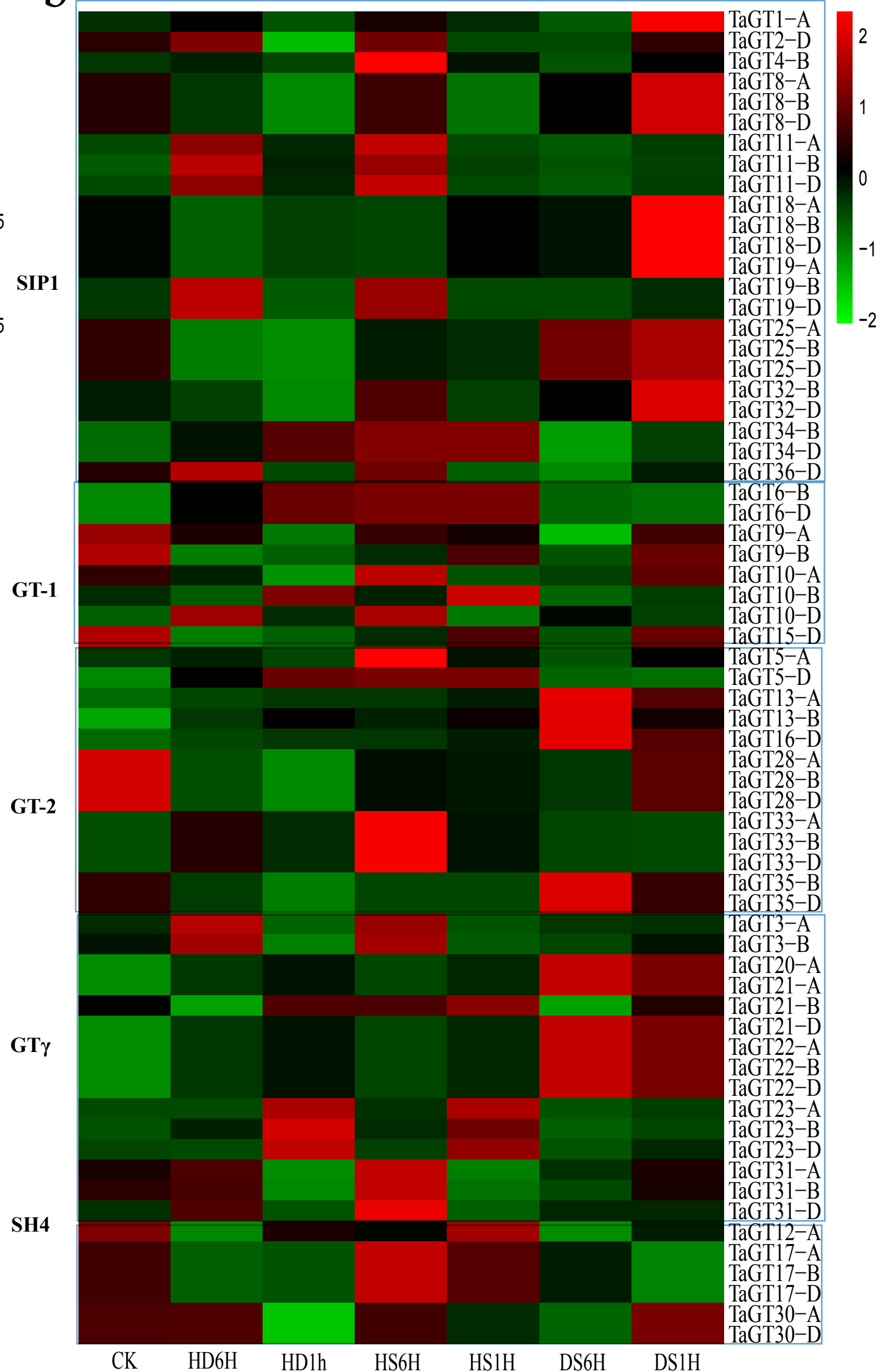

Supplement: Supplementary file 29 — Expression patterns of TaGT genes in various wheat tissues (a) and stresses (b).The expression data was collected through pubic RNA-seq data. The tissues expression of TaGT genes at different growth stages, such as roots (cotyledon emergence stage), stems (FL.02 1/2 of flowers open stage) leaves tissues (cotyledon emergence stage) and flowers (FL.02 1/2 of flowers open stage). Heatmap of expression profiles for TaGT genes across different stresses under 1 and 6 h’s treatments, including heat stress, drought stress and drought&heat combined stress. (PDF 461 kb) [file 12864_2019_5494_MOESM29_ESM.pdf]

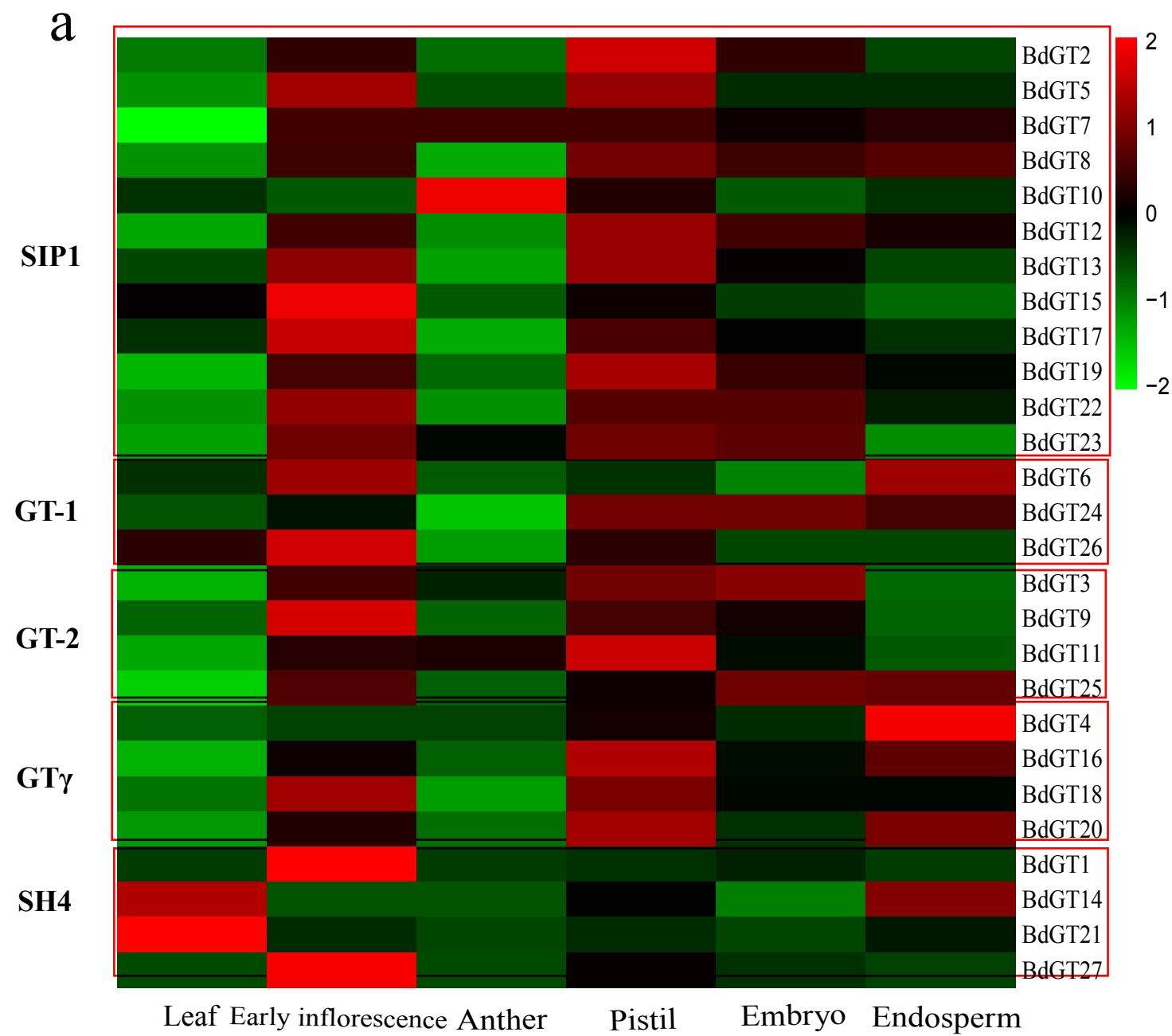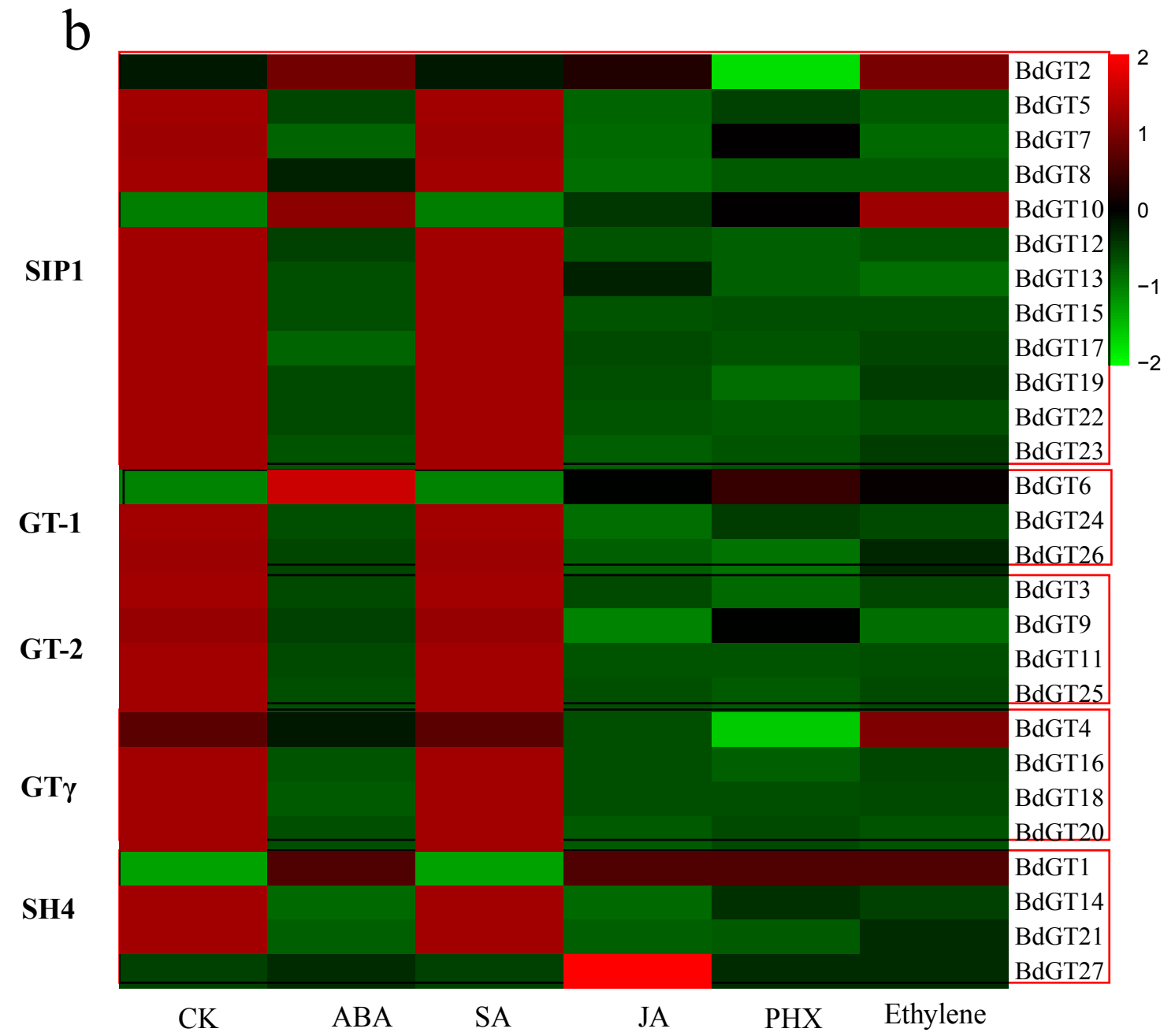

Supplement: Supplementary file 30 — Expression patterns of BdGT genes in various B. distachyon (a) organs (Leaf, inflorescence, anther, pistil, plant embryo, and endosperm) and abiotic stresses (CK, ABA, SA, JA, PHX, and Ethylene). (PDF 364 kb) [file 12864_2019_5494_MOESM30_ESM.pdf]

**a**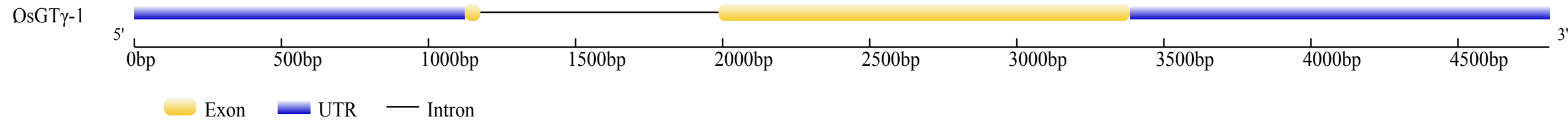**b**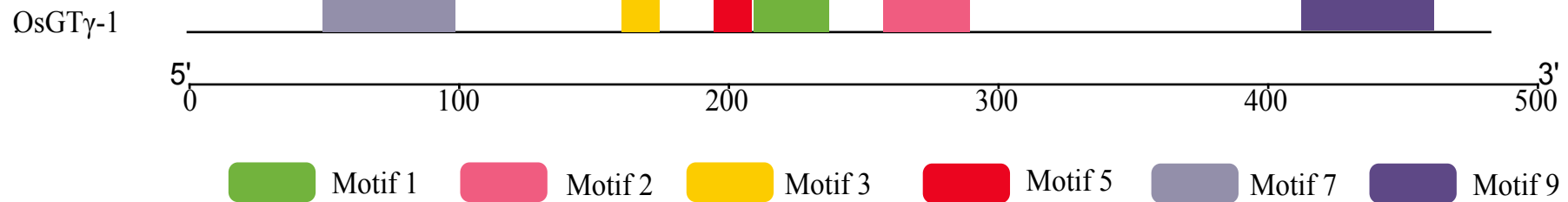

Supplement: Supplementary file 32 — Gene structure and motifs of .OsGTγ-1 protein. In the gene structure part, blue boxes represent UTRs, yellow boxes represent exons, and black lines represent introns. In the motif part, the boxes in different color represent different motifs, and the black lines represent un-conserved sequences. (PDF 254 kb) [file 12864_2019_5494_MOESM32_ESM.pdf]
