# Supplementary material for: Comparison of Trihelix transcription factors between wheat and Brachypodium distachyon at genome-wide
Source: BMC Genomics. 2019 Feb 15;20:142. doi: 10.1186/s12864-019-5494-7 (PMC6377786; doi:10.1186/s12864-019-5494-7)
Supplement: Supplementary file 5 — Multiple sequence alignment of GT domains of wheat and B. distachyon Trihelix TFs. Helix 1, 2 and 3 rectangular bars represent trihelix structure identified in the GT domain. Yellow shade indicates conserved amino acids. (PDF 6136 kb) [file 12864_2019_5494_MOESM5_ESM.pdf]

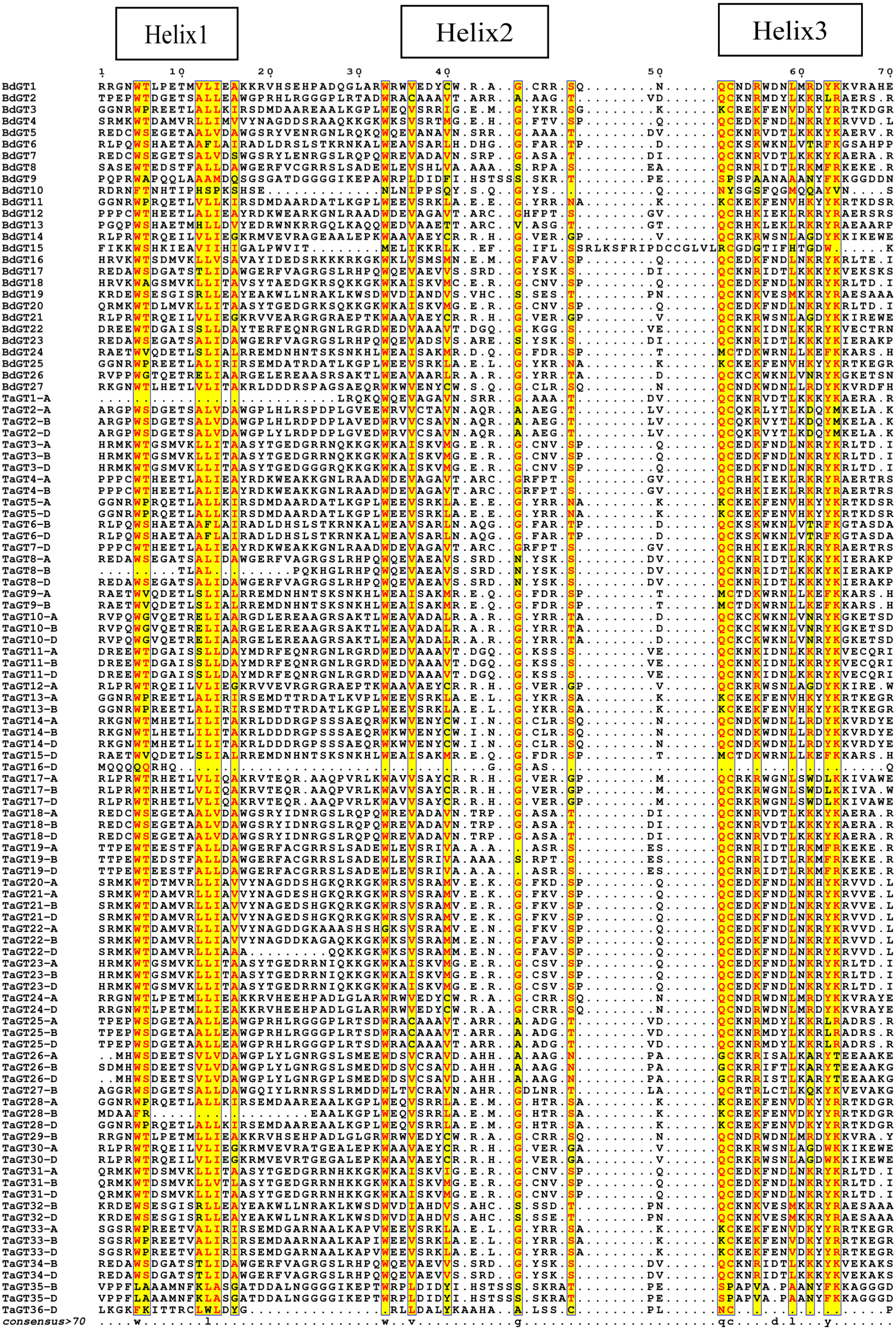
Helix1
Helix2
Helix3
